# TASC-SwinMT: Task-Adaptive Synergistic Cross-Task Swin Multi-Task Framework for CT and MRI Image Interpolation and Segmentation

**DOI:** 10.3390/tomography12060080

**Published:** 2026-05-30

**Authors:** Yujia Sun, Yingying Yang, Nan Bao

**Affiliations:** School of Biomedical Engineering, Northeastern University, Shenyang 110169, China; sunyujia@mails.neu.edu.cn (Y.S.); yangyy2@mails.neu.edu.cn (Y.Y.)

**Keywords:** Computed Tomography, Magnetic Resonance Imaging, multi-task learning, image interpolation, medical image segmentation, Swin Transformer, task-aware adapter, cross-task interaction, feature alignment fusion

## Abstract

**Background:** Computed Tomography (CT) and Magnetic Resonance Imaging (MRI) interpolation and segmentation are critical for clinical diagnosis, anatomical quantification and personalized treatment. Most existing methods perform these two tasks separately, leading to computational redundancy and insufficient mining of shared spatial features. This study aims to construct an integrated multi-task learning framework for the synchronous processing of medical image interpolation and segmentation. **Methods:** We propose a unified multi-task framework named TASC-SwinMT for joint interpolation and multi-frame segmentation of CT and MRI images. It employs a shared SwinUNet encoder to extract general spatial features, matched with two task-specific decoders for frame prediction and mask generation. Three functional modules are designed for cross-task synergistic learning, and a dynamic multi-task loss function is used to balance objective optimization. Experiments are performed on Medical Segmentation Decathlon Task02_Heart and Task06_Lung datasets. **Results:** Our method outperforms baseline models and ablation variants in both tasks with outstanding accuracy and significantly reduced computational overhead. It exhibits superior performance in lesion boundary depiction, small object segmentation and inter-slice consistency, and anatomical prior constraints with frequency-domain modeling further enhance prediction quality. **Conclusions:** The cross-task feature sharing and joint optimization strategy are validated effective. The proposed TASC-SwinMT framework has favorable stability and generalization ability, providing a reliable solution for clinical medical image analysis.

## Simple Summary

Medical CT and MRI image interpolation and segmentation are essential for clinical thoracic disease diagnosis and anatomical analysis, yet most existing methods handle these two tasks separately with redundant computation and insufficient cross-task feature mining. This study proposes TASC-SwinMT, a unified task-adaptive synergistic cross-task Swin multi-task framework for joint interpolation and segmentation. Three dedicated collaborative modules and a learnable dynamic loss are designed to enable adaptive feature modulation, fine-grained cross-task interaction, and balanced joint optimization. Evaluated on public heart MRI and lung CT datasets, TASC-SwinMT outperforms mainstream baseline and state-of-the-art models in both interpolation reconstruction and lesion segmentation, while substantially reducing computational overhead. The proposed framework provides an efficient, generalizable solution for synchronous medical image interpolation and segmentation, holding practical application potential in routine clinical thoracic imaging analysis.

## 1. Introduction

CT and MRI serve as key imaging tools for the diagnosis and treatment planning of cardiovascular and thoracic diseases [1,2,3]. Lung tumor and left atrium segmentation support precise anatomical localization and volumetric quantification, and slice interpolation resolves axial resolution anisotropy, supplements missing inter-slice anatomical details and enhances temporal consistency of clinical analysis [4,5,6]. Interpolation and segmentation share inherent spatiotemporal correlation and complementary anatomical attributes in medical image post-processing, making their joint optimization a valuable research direction [7,8,9].

Existing studies usually take CT interpolation and segmentation as independent single-task pipelines [10,11,12], which brings two obvious limitations. Independent network design produces redundant computation, as repeated extraction of low-level spatial and high-level semantic features increases computational cost [13,14,15]. Insufficient cross-task interaction also restricts model performance. Interpolation without anatomical constraints from segmentation may produce clinically unrealistic structures, and segmentation lacking spatiotemporal context from interpolation leads to inaccurate small-target detection and poor inter-slice consistency [16,17,18]. In addition, Convolutional Neural Network(CNN) and Transformer single-task models cannot effectively capture long-range spatiotemporal dependencies and adapt to task-specific feature requirements for chest CT and cardiac MRI analysis [19,20,21].

With the advance of medical deep learning, CNN-based U-Net variants [22,23,24] had achieved SOTA segmentation performance. Swin Transformer-based models [25,26,27] overcome CNN limitations in long-range dependency modeling and show great potential in CT reconstruction and segmentation [28,29,30]. Multi-task learning (MTL) has also been widely used in medical image analysis, including joint segmentation-classification [31,32,33] and joint detection-segmentation [34,35], validating feature sharing and mutual promotion between correlated tasks [36,37,38]. However, current MTL methods rarely focus on joint interpolation-segmentation for chest CT and cardiac MRI; relevant works only adopt simple feature concatenation or weighted fusion [39,40,41], lacking task-adaptive feature modulation, fine-grained cross-task interaction and dynamic optimization balance for heterogeneous objectives [20,42,43].

To tackle these problems, this work proposes TASC-SwinMT, a Task-Adaptive Synergistic Cross-Task Swin Multi-Task framework for joint interpolation and segmentation of CT and MRI. The initial four-letter acronym TASC corresponds to the core design concept of Task-Adaptive modeling, Synergistic learning, Cross-task correlation and task-oriented optimization. The latter part of SwinMT corresponds to the Swin Transformer backbone and the Multi-Task learning paradigm adopted in the overall framework. Leveraging spatiotemporal correlation between the two tasks, the framework builds a Swin Transformer-based collaborative architecture for efficient feature sharing and performance mutual promotion. We adopt a shared SwinUNet encoder to extract universal spatial features from CT and MRI inputs, paired with two task-specific decoders for interpolated frame generation and multi-frame segmentation. Three dedicated multi-task modules are designed: TALA integrates spatial dual-bottleneck extraction and frequency-domain global modeling for task-adaptive feature embedding; MSTAF realizes cross-level feature alignment via bidirectional cross-attention, multi-scale fusion and frequency enhancement; CTCI achieves fine-grained cross-task feature interaction through multi-scale spatial extraction, frequency alignment and dynamic gating. Additionally, a learnable dynamic multi-task loss is proposed to balance pixel-level interpolation reconstruction and segmentation classification, avoiding training bias toward single tasks.

Extensive experiments on Medical Segmentation Decathlon (MSD) Task02_Heart and Task06_Lung datasets [44] show TASC-SwinMT outperforms mainstream single-task baseline models and ablation variants on both interpolation and segmentation tasks. The framework improves lesion boundary fidelity and small-target segmentation accuracy, enhances inter-slice temporal consistency, and provides a robust generalizable solution for clinical CT and MRI analysis.

The main contributions of this paper are summarized as follows:

1. We propose TASC-SwinMT, a unified multi-task framework for joint interpolation and segmentation of CT and MRI, which exploits task spatiotemporal correlation and anatomical complementarity to realize feature sharing and performance improvement.
2. Three customized multi-task collaboration modules are developed: TALA enables task-adaptive modulation via spatial dual-bottleneck and frequency-domain modeling; MSTAF implements cross-level feature alignment; CTCI achieves fine-grained adaptive cross-task interaction. These modules jointly mitigate feature deficiency and distribution mismatch in multi-task learning.
3. A learnable dynamic multi-task loss is designed to balance heterogeneous optimization objectives of interpolation and segmentation, with better flexibility and effectiveness than traditional fixed-weight loss strategies.
4. Experiments on public MSD datasets verify the superiority of TASC-SwinMT over mainstream single-task and multi-task baseline methods. Ablation studies further confirm the effectiveness of each core module embedded in the proposed framework.

The remainder of this paper is structured as follows: Section 2 reviews related work on medical interpolation, segmentation, multi-task learning and Swin Transformer. Section 3 introduces datasets, detailed framework architecture and training settings. Section 4 presents experimental results and analysis. Section 5 discusses results, limitations and future directions. Section 6 draws the final conclusion.

## 2. Related Work

This section summarizes core research progress of medical image interpolation, medical image segmentation, multi-task learning and Swin Transformer-based medical vision methods. This section also clarifies inherent limitations of existing studies and identifies the research gaps targeted by the proposed framework.

### 2.1. Medical Image Interpolation

Medical CT interpolation generates intermediate slice frames to enhance volumetric spatial resolution and supplement missing anatomical details in tomographic imaging [45,46,47]. Traditional interpolation methods adopt linear fitting, B-spline transformation and Fourier transformation to complete slice reconstruction [9,48]. These methods maintain stable computational efficiency but cannot fit complex anatomical structures well, and they tend to produce blurred textures and inconsistent spatial structures in lesion-rich regions [4,49].

Deep learning approaches have become the mainstream technical scheme for medical slice interpolation in recent years [5,6,8]. Representative studies construct dedicated network frameworks to capture non-rigid motion features and cross-view texture correlation for cardiac MRI and anisotropic CT interpolation [4,5,7]. Other research directions combine interpolation strategy with sparse-view and low-dose CT reconstruction to improve clinical imaging quality [2,29,30,39].

Current deep learning interpolation methods still have two obvious defects in practical application. Most existing models work under single-task settings without introducing anatomical prior information from segmentation tasks [13,50,51]. Few studies introduce frequency-domain feature modeling to enhance global spatiotemporal representation, which restricts reconstruction fidelity of lesion boundaries. This work embeds segmentation constraint information and frequency-domain global modeling into interpolation feature learning to remedy the above deficiencies.

### 2.2. Medical Image Segmentation

Medical image segmentation serves as the core technical support for anatomical localization and pathological lesion quantification in clinical diagnosis [17,52,53]. CNN-based U-Net and its derivative variants lay the foundation for medical segmentation due to powerful local feature extraction and semantic transmission capability [22,23,24,25,26,27,54]. Improved U-Net structures are continuously proposed to adapt to abdominal organ segmentation, paranasal sinus CT analysis and other clinical scenarios [18,55].

Transformer-based segmentation models break the long-range dependency modeling bottleneck of traditional CNN structures [12,25,26,27]. Series Transformer frameworks including UNETR and Swin-Unet achieve state-of-the-art performance on public medical segmentation datasets [26,27]. Many studies further integrate CNN and Transformer structures to obtain complementary advantages for tumor and organ segmentation tasks [15,21,53].

### 2.3. Multi-Task Learning in Medical Image Analysis

Multi-task learning realizes simultaneous optimization of multiple correlated tasks through shared feature extraction, and it can reduce computational redundancy while improving model generalization ability [36,37,38]. This learning paradigm has been widely applied to multiple medical analysis scenarios including joint segmentation and classification, joint segmentation and detection, and joint reconstruction and classification [31,32,33,34,35,56,57,58].

Existing multi-task learning methods have achieved satisfactory results in CT reconstruction, pathological classification and multi-modal feature fusion [39,40,41,43,57,58]. Three key research gaps still remain for joint interpolation and segmentation of CT and MRI. Few existing works focus on joint optimization of the two tasks and fail to fully mine cross-task complementary correlation [39,40]. Most multi-task frameworks adopt simple feature concatenation and lack fine-grained interactive fusion and task-adaptive modulation design [20,42,59]. Fixed weight loss functions cannot balance heterogeneous optimization objectives and easily cause training bias toward single tasks [43,60,61]. The proposed framework fills these gaps through task-aware feature modulation, bidirectional cross-task interaction and dynamic loss weight optimization.

### 2.4. Swin Transformer for Medical Computer Vision

Standard Vision Transformer encounters high computational complexity and poor adaptability for high-resolution medical tomographic images [25,26,27,62]. Swin Transformer adopts hierarchical structure and shifted window self-attention to reduce computational complexity, and it has been widely used in medical feature extraction and downstream task modeling [10,11,26].

Swin Transformer-based models dominate segmentation performance on mainstream medical datasets [10,26,27]. Relevant research also extends Swin Transformer to medical image interpolation and low-dose CT denoising tasks [39,40,50]. Preliminary attempts combine Swin Transformer with multi-task learning for histological image analysis [20,42].

Two limitations restrict the application of Swin Transformer in thoracic image multi-task learning. Most Swin-based networks are designed for single-task scenarios and do not support task-specific feature adaptation in multi-task settings [14,15,63]. Frequency-domain modeling is rarely integrated into Swin Transformer feature learning, which weakens global structural perception and lesion boundary representation ability [13,50,51]. This work constructs a shared Swin Transformer encoder with dedicated task-aware modules and embeds frequency-domain modeling to solve the above problems.

### 2.5. Summary of Related Methods

To further clarify the differences between existing representative methods and the proposed framework, key technical features, applicable scenarios and inherent limitations of typical related works are summarized in Table 1.

## 3. Materials and Methods

This section elaborates on the dual public datasets Medical Segmentation Decathlon (MSD) Task06_Lung and Task02_Heart. This data can be accessed at: http://medicaldecathlon.com/dataaws/ (accessed on 5 March 2026). The detailed architecture of the proposed TASC-SwinMT model, the comprehensive training and evaluation configuration, and the definition of key symbols involved in the framework are presented. All implementations are built on the PyTorch deep learning framework with Graphics Processing Unit (GPU) acceleration (NVIDIA RTX 4090, 24 GB VRAM) to ensure efficient model training and inference. The source code of this work is publicly available at GitHub: https://github.com/yujiasun88-hash/TASC (accessed on 16 May 2026).

### 3.1. Datasets

To validate the generalization ability of TASC-SwinMT across different anatomical structures, we conduct experiments on two representative public datasets from the Medical Segmentation Decathlon (MSD), which are widely used and standardized in medical image analysis.

#### 3.1.1. MSD Task06_Lung Dataset

The MSD Task06_Lung dataset is a publicly available thoracic CT dataset with voxel-level annotations of lung tumors that contains clinical cases with diverse lesion morphology, size, and location, making it suitable for evaluating both interpolation fidelity and tumor-aware segmentation performance in challenging pulmonary regions.

Sample construction follows a triplet-based strategy. We select two input frames with an interval of 2.5 mm as inputFrame1 and inputFrame2. These two frames are used to generate an interpolated middle frame with an interval of 1.25 mm. The three frames form a single training unit, where the interpolated frame acts as the ground-truth target for the interpolation task. We perform segmentation on the two original input frames and the reconstructed interpolated frame to ensure temporal consistency of lesion representation. We divide the whole dataset into training, validation, and test subsets following a fixed 7:1:2 partitioning ratio. All image samples collected from the same patient are assigned exclusively to one single subset at the patient level rather than the slice level. This patient-wise partitioning rule avoids potential data leakage and guarantees the validity of subsequent experimental results. We also remove invalid samples with insufficient consecutive slices to maintain overall data quality.

#### 3.1.2. MSD Task02_Heart Dataset

The MSD Task02_Heart dataset is a publicly available thoracic MRI dataset with voxel-level annotations for left atrium regions. Compared with lung lesion segmentation, the left atrium possesses clearer boundary contrast and suffers less tissue interference, leading to lower task difficulty. It serves as a complementary testbed to verify the robustness of the proposed multi-task framework across anatomically distinct organs.

The sample construction strategy is consistent with that of the Lung dataset. We select two input frames with an interval of 2.5 mm to generate an interpolated middle frame with an interval of 1.25 mm. The two original input frames and the reconstructed interpolated frame form a unified unit for joint interpolation and segmentation tasks. We adopt the identical 7:1:2 ratio to partition the heart dataset into training, validation, and test sets. The partitioning is implemented strictly at the patient level to ensure all slices from one patient belong only to a single subset. This standard protocol eliminates slice-level random splitting and prevents data leakage during model training and evaluation. Strict data screening is conducted to exclude invalid samples and secure reliable experimental outcomes of the TASC-SwinMT model.

#### 3.1.3. Unified Data Preprocessing

To eliminate potential biases caused by dataset-specific differences and ensure fair comparison between models, we adopted a consistent data preprocessing pipeline for both datasets. This standardized procedure offsets inherent gaps in intensity distributions and acquisition characteristics existing between CT and MRI modalities, and it includes the following steps:

1. Data format conversion: Read the nii.gz files using the nibabel library and convert them into PyTorch tensors with the dimension order Batch×Channels×Height×Width to adapt to the input requirements of deep learning models.
2. Pixel value normalization: Perform per-image normalization to scale pixel values into the range [0, 1]. This step eliminates the impact of differences in scanning devices and imaging parameters, and unifies data distribution characteristics of input CT and MRI images.
3. Training set data augmentation: To mitigate overfitting, enhance model generalization and robustness to imaging noise, we applied a comprehensive data augmentation strategy to the training set. The augmentation operations include random horizontal and vertical flipping with a probability of 0.5, random rotation within the range of $0^{\circ }$ to $15^{\circ }$, random scaling with factors ranging from 0.9 to 1.1, and additive Gaussian noise injection (mean = 0, standard deviation = 0.01) to simulate real-world medical imaging noise. For the validation and test sets, only format conversion and normalization are performed without any augmentation to reflect the true performance of the model on unseen data.

### 3.2. Proposed Model

The proposed TASC-SwinMT model is specifically designed for joint interpolation and segmentation of thoracic CT and MRI images, focusing on lung tumor segmentation with interpolation and left atrium segmentation with interpolation. The model takes as input two consecutive CT/MRI frames, denoted as $X_{1},X_{3}\in R^{B\times 1\times H\times W}$, and outputs the interpolated middle frame ${\hat{X}}_{2}\in R^{B\times 1\times H\times W}$ and segmentation masks for the three frames, denoted as ${\hat{Y}}_{1},{\hat{Y}}_{2},{\hat{Y}}_{3}\in R^{B\times 1\times H\times W}$. The segmentation masks correspond to lung tumors for lung CT inputs and left atrium for heart MRI inputs. The complete forward propagation process of the proposed TASC-SwinMT model is summarized in Algorithm 1.

**Figure 1:**
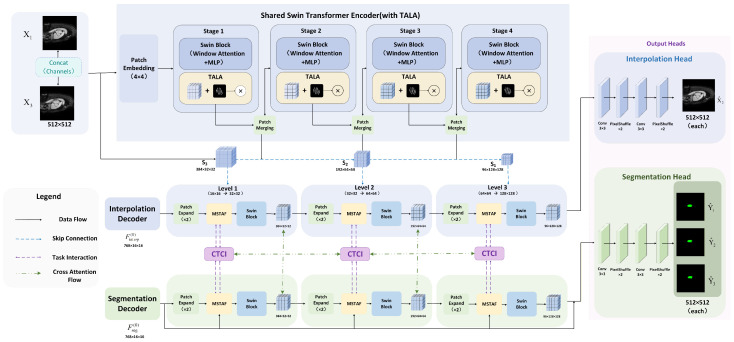
Overall framework of TASC-SwinMT.

The model follows five core design principles, as illustrated in Figure 1:

1. The Task-Aware Lightweight Adapter (TALA) extracts spatial dual-bottleneck features and global frequency-domain information, and injects task-adaptive features into the shared encoder.
2. Multi-Scale Task Alignment Fusion (MSTAF) uses bidirectional cross-attention, multi-scale spatial extraction, and frequency-domain enhancement to align encoder-decoder skip connections.
3. Cross-Task Collaborative Interaction (CTCI) enables adaptive fine-grained feature interaction between interpolation and segmentation tasks.
4. The shared encoder and dual decoders balance universal feature sharing and task-specific learning to reduce redundancy.
5. A dynamic multi-task loss with learnable weights balances heterogeneous task optimization and avoids single-task dominance.

#### 3.2.1. Task-Aware Lightweight Adapter (TALA)

**Figure 2:**
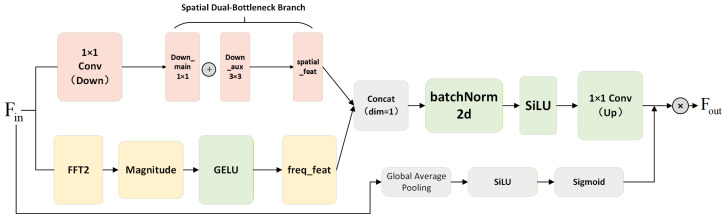
Structure of the Task-Aware Lightweight Adapter.

To address the lack of task specificity in shared encoder features and capture global frequency information, we propose the Task-Aware Lightweight Adapter. This module extracts spatial dual-bottleneck features and performs frequency-domain global modeling to generate task-adaptive representations. Embedded dynamic channel attention gating generates modality adaptive feature representations, and enables the shared encoder to extract stable anatomical features from incoming CT and MRI images. The structure of the proposed TALA module is shown in Figure 2.

The mathematical formulation of TALA is defined as follows:

**Step 1: Spatial Dual-Bottleneck Feature Extraction**

Given input feature $F_{in}\in R^{B\times C\times H\times W}$, a 1×1 convolution is first used to reduce channel dimension.

(1) $$\begin{array}{cc} F_{down} & ={\mathrm{Conv}}_{1\times 1}\left(F_{in}\right),\\ F_{main} & ={\mathrm{Conv}}_{1\times 1}\left(F_{down}\right),\\ F_{aux} & ={\mathrm{Conv}}_{3\times 3}\left(F_{down}\right),\\ F_{spatial} & =F_{main}+F_{aux} \end{array}$$

The dual-bottleneck structure efficiently extracts local spatial details with limited computational cost.

**Step 2: Frequency-Domain Global Feature Modeling**

Global structural information is captured by transforming features into the frequency domain using two-dimensional fast Fourier transform (FFT).

(2) $$\begin{array}{cc} F_{fft} & =\mathrm{FFT}2(F_{in}),\\ F_{mag} & =|F_{fft}|,\\ F_{freq} & =\mathrm{GELU}(F_{mag}) \end{array}$$

This branch provides long-range global context that complements local spatial features.

**Step 3: Cross-Modal Feature Fusion**

Spatial and frequency features are concatenated and normalized for unified representation.

(3) $$\begin{array}{cc} F_{concat} & =\mathrm{Concat}(F_{spatial},F_{freq},\dim=1),\\ F_{bn} & ={\mathrm{BN}}_{2d}\left(F_{concat}\right),\\ F_{silu} & =\mathrm{SiLU}(F_{bn}),\\ F_{fusion} & ={\mathrm{Conv}}_{1\times 1}\left(F_{silu}\right) \end{array}$$

**Step 4: Dynamic Channel Attention Gating**

A channel attention mechanism generates adaptive weights to emphasize task-relevant features.

(4) $$\begin{array}{cc} F_{gap} & =\mathrm{AdaptiveAvgPool}2d(F_{in}),\\ F_{att} & =\mathrm{SiLU}(F_{gap}),\\ \alpha & =\mathrm{Sigmoid}(F_{att}),\\ F_{out} & =F_{fusion}⊗\alpha \end{array}$$

The attention weight $\alpha_{\mathrm{tala}}$ dynamically adjusts feature channels according to task requirements. The final output $F_{out}$ maintains the same dimension as the input and provides task-adaptive enhanced features for subsequent modules.

#### 3.2.2. Multi-Scale Task Alignment Fusion (MSTAF)

**Figure 3:**
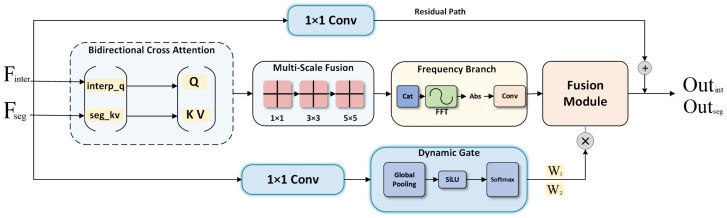
Structure of the multi-scale task alignment fusion.

To address feature distribution mismatch and spatial misalignment between encoder skip connection features and decoder upsampling features, we propose the Multi-Scale Task Alignment Fusion module. This module integrates bidirectional cross-attention, multi-scale spatial fusion, frequency-domain enhancement and dynamic gating to achieve precise feature alignment and adaptive fusion. The structure of the proposed MSTAF module is shown in Figure 3.

The complete mathematical formulation is defined as follows:

(5) $$\begin{array}{cc} Q_{1} & =W_{q}^{\left(1\right)}\cdot \mathrm{Flatten}\left(F_{inter}\right)+b_{q}^{\left(1\right)},\\ K_{1},V_{1} & =W_{kv}^{\left(1\right)}\cdot \mathrm{Flatten}\left(F_{seg}\right)+b_{kv}^{\left(1\right)},\\ Q_{2} & =W_{q}^{\left(2\right)}\cdot \mathrm{Flatten}\left(F_{seg}\right)+b_{q}^{\left(2\right)},\\ K_{2},V_{2} & =W_{kv}^{\left(2\right)}\cdot \mathrm{Flatten}\left(F_{inter}\right)+b_{kv}^{\left(2\right)},\\ A_{1} & =\mathrm{Softmax}\left(\frac{K_{1}^{⊤}Q_{1}}{\sqrt{d_{k}}}\right)V_{1},\\ A_{2} & =\mathrm{Softmax}\left(\frac{K_{2}^{⊤}Q_{2}}{\sqrt{d_{k}}}\right)V_{2},\\ F_{att} & ={\mathrm{Conv}}_{1\times 1}\left(\mathrm{Concat}\left(A_{1},A_{2}\right)\right),\\ F_{1\times 1} & ={\mathrm{Conv}}_{1\times 1}\left(F_{att}\right),\\ F_{3\times 3} & ={\mathrm{Conv}}_{3\times 3}\left(F_{att}\right),\\ F_{5\times 5} & ={\mathrm{Conv}}_{5\times 5}\left(F_{att}\right),\\ F_{multi} & ={\mathrm{Conv}}_{1\times 1}\left(\mathrm{Concat}\left(F_{1\times 1},F_{3\times 3},F_{5\times 5}\right)\right),\\ F_{fft} & =\mathrm{FFT}2D(F_{multi}),\\ M & =\mathrm{Abs}\left(F_{fft}\right),P=∠F_{fft},\\ M^{\prime } & ={\mathrm{Conv}}_{1\times 1}\left(M\right),\\ F_{freq} & =\mathrm{iFFT}2D(M^{\prime }\cdot e^{jP}),\\ F_{gate} & ={\mathrm{Conv}}_{1\times 1}\left(F_{seg}\right),\\ g & =\mathrm{GlobalPool}(F_{gate}),\\ g^{\prime } & =\mathrm{SiLU}(g),\\ {\left[W_{1},W_{2}\right]}^{⊤} & =\mathrm{Softmax}(W_{\mathrm{soft}}g^{\prime }+b_{\mathrm{soft}}),\\ F_{freq}^{\left(inter\right)} & =F_{freq},\;F_{freq}^{\left(seg\right)}=F_{freq},\\ F_{fusion} & =W_{1}F_{freq}^{\left(inter\right)}+W_{2}F_{freq}^{\left(seg\right)},\\ F_{res} & ={\mathrm{Conv}}_{1\times 1}\left(F_{inter}\right),\\ F_{res}^{\left(seg\right)} & ={\mathrm{Conv}}_{1\times 1}\left(F_{seg}\right),\\ {\mathrm{Out}}_{int} & =F_{fusion}+F_{res},\\ {\mathrm{Out}}_{seg} & =F_{fusion}+F_{res}^{\left(seg\right)}. \end{array}$$

The module takes interpolation feature $F_{inter}$ and segmentation feature $F_{seg}$ as inputs. Bidirectional cross-attention is first applied to model the interdependency between the two task features. Multi-scale convolutions extract spatial features with various receptive fields. The frequency branch enhances fine-grained details through fast Fourier transform. Dynamic gating generates adaptive fusion weights for the two tasks. The fused features are combined with residual connections to produce the final outputs for interpolation and segmentation tasks.

The decoder upsampling feature is denoted as $f_{dec}\in R^{B\times C\times H\times W}$. The encoder skip connection feature is denoted as $f_{skip}$. The bidirectional cross-attention mechanism enables mutual guidance between interpolation and segmentation features. Multi-scale spatial fusion captures comprehensive contextual information. The frequency-domain enhancement branch improves the representation of fine structures and boundaries. Dynamic gating adaptively adjusts the contribution of each task feature. Residual connections preserve original information and stabilize network training.

#### 3.2.3. Window Attention in Swin Transformer

To efficiently model global context for 512×512 CT images, we adopt fixed window self-attention with shifted windows in the Swin Transformer backbone. The core components are:

1. Window Partition
  (6) windows=Partition(F,win_size=8)
2. Query-Key-Value Projection
  (7) $$\begin{array}{cc} Q,K,V & =\mathrm{Linear}(F)\to 3\times C\\ \mathrm{attn} & =\mathrm{Softmax}\left(\frac{QK^{T}}{\sqrt{d_{k}}}\right)V\\ F_{attn} & =\mathrm{Linear}(\mathrm{attn}) \end{array}$$

where $d_{k}=C/N_{h}$ is the head dimension, $N_{h}$ is the number of attention heads, and shifted windows eliminate boundary artifacts.

3. MLP Feature Refinement
  (8) $$F_{mlp}=\mathrm{Linear}\left(\mathrm{GELU}\left(\mathrm{Linear}\left(F_{attn},C\to 4C\right)\right)\right)$$

#### 3.2.4. SwinTransformerBlock with TALA

The SwinTransformerBlock serves as the core feature extraction unit, integrating window attention, MLP, and residual connections for medical image feature learning:

(9) $$\begin{array}{cc} F_{norm1} & =\mathrm{LayerNorm}(F),\\ F_{attn} & =\mathrm{WindowAttention}(F_{norm1})⊕F,\\ F_{norm2} & =\mathrm{LayerNorm}(F_{attn}),\\ F_{mlp} & =\mathrm{MLP}\left(F_{norm2}\right)⊕F_{attn},\\ F_{tala} & =\mathrm{TALA}(F_{mlp}),\\ F_{out} & =F_{tala}⊕F_{mlp}. \end{array}$$

The TALA module is inserted after each encoder block to inject task-adaptive features with spatial and frequency-domain modeling while preserving shared universal feature representations.

#### 3.2.5. Cross-Task Collaborative Interaction (CTCI) Module

**Figure 4:**
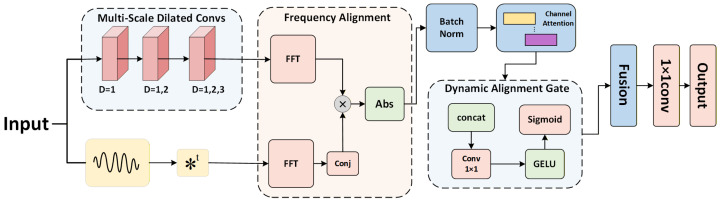
Structure of the cross-task collaborative interaction.

To realize bidirectional feature guidance between interpolation and segmentation tasks, we design the CTCI module with multi-scale spatial feature extraction, frequency-domain alignment, channel attention and dynamic alignment gating. This module establishes accurate cross-task feature correlation and achieves adaptive interaction between heterogeneous task features. The structure of the proposed CTCI module is shown in Figure 4.

Given the interpolation feature $F_{interp}$ and segmentation feature $F_{seg}$, the CTCI module first extracts multi-scale spatial features through cascaded dilated convolutions. The temporal branch performs cross-correlation operation for preliminary feature alignment. The frequency alignment module converts features into the frequency domain and computes cross-correlation to achieve global feature matching. Batch normalization (BN) stabilizes the feature distribution, and channel attention enhances task-relevant channels. Dynamic alignment gating generates adaptive weights to refine the aligned features. Notably, the symbol ∗ in the figure represents element-wise multiplication (Hadamard product). The mathematical formulation is defined as follows:  

(10) $$\begin{array}{cc} X_{s} & =\mathrm{MultiScaleDilatedConv}(F_{interp}),\\ X_{t} & =\mathrm{TempCrossCorr}(F_{interp},F_{seg}),\\ F_{\mathrm{align}} & =|F\left(X_{s}\right)⊙\bar{F(X_{t})}|,\\ F_{\mathrm{bn}} & =\mathrm{BN}(F_{\mathrm{align}}),\\ F_{\mathrm{ca}} & =\mathrm{ChannelAttention}(F_{\mathrm{bn}}),\\ F_{\mathrm{cat}} & =F_{\mathrm{ca}}⊕F_{\mathrm{align}},\\ G & =\sigma \left(\mathrm{GELU}({\mathrm{Conv}}_{1\times 1}\left(F_{\mathrm{cat}}\right))\right),\\ F_{\mathrm{gate}} & =G⊙F_{\mathrm{align}},\\ F_{interp}^{*},F_{seg}^{*} & ={\mathrm{Conv}}_{1\times 1}\left(F_{\mathrm{gate}}\right) \end{array}$$

Multi-scale dilated convolutions capture spatial context at different scales. Frequency alignment eliminates spatial and temporal misalignment between cross-task features. Channel attention and dynamic alignment gating adaptively enhance informative features and suppress redundant information. The module promotes collaborative representation learning between interpolation and segmentation tasks.

#### 3.2.6. Patch Embedding/Merging/Expand Modules

##### PatchEmbed (Input Projection)

Converts raw CT images to patch embeddings via convolution:

(11) $$F_{0}={\mathrm{Conv}}_{4\times 4,\mathrm{stride}=4}\left(X\right)$$

where $F_{0}\in R^{B\times 96\times 128\times 128}$.

##### PatchMerging (Downsampling)

Spatial downsampling with channel doubling for encoder:

(12) $$\begin{array}{cc} F_{merge} & =\mathrm{concat}(F_{0::2},F_{1::2},F_{0::2},F_{1::2})\\ F_{down} & =\mathrm{Linear}(\mathrm{LayerNorm}\left(F_{merge}\right)) \end{array}$$

where $F_{down}\in R^{B\times 2C\times H/2\times W/2}$.

##### PatchExpand (Upsampling)

PatchExpand performs spatial upsampling and channel reduction for the decoder path.

(13) $$\begin{array}{cc} F_{expand} & =\mathrm{Linear}(F),\\ F_{up} & =\mathrm{Reshape}(\mathrm{Permute}\left(F_{expand}\right),B,C/2,2H,2W) \end{array}$$

#### 3.2.7. Shared Encoder

The shared encoder extracts universal spatial features from concatenated input frames, enabling efficient feature sharing between tasks. The encoder possesses strong adaptability to different imaging modalities, and completes unified feature extraction for input CT and MRI data. The input to the encoder is the concatenation of two consecutive imaging frames $X=\mathrm{Concat}\left(X_{1},X_{3}\right)\in R^{B\times 2\times 512\times 512}$. The encoder processing pipeline is:

1. Patch embedding: Generate 96-channel feature map $F_{0}\in R^{B\times 96\times 128\times 128}$.
2. Four-stage feature extraction: Each stage consists of:
  - SwinTransformerBlock with shifted window attention;
  - TALA module for task-aware, frequency-aware and modality-aware feature enhancement;
  - PatchMerging (except the 4th stage) for spatial downsampling.

The detailed evolution of feature dimensions across different encoder stages remains consistent with Table 2:

#### 3.2.8. Dual Decoders with CTCI

We design two symmetric dedicated decoders for interpolation and segmentation, integrated with MSTAF and CTCI modules for cross-task and cross-modality collaboration:

Each decoder includes three upsampling stages. Each stage contains:

1. Upsampling: PatchExpand module to match the spatial resolution of encoder skip connections.
2. Skip Connection Fusion: MSTAF aligns decoder features and encoder features in the frequency domain, and eliminates feature deviation brought by different imaging modalities.
3. Feature Refinement: SwinTransformerBlock for feature optimization.
4. Cross-Task Feature Interaction: CTCI module fuses interpolation and segmentation features with bidirectional attention guidance, and maintains stable prediction performance on diverse CT and MRI imaging data.

#### 3.2.9. Output Heads with PixelShuffle

##### Interpolation Output Head

Generates interpolated CT frames via dual sub-pixel upsampling:

(14) $$\begin{array}{cc} F_{up1} & =\mathrm{PixelShuffle}(\mathrm{Conv}3\times 3\left(F_{interp},C\to 4C\right)),\\ F_{up2} & =\mathrm{PixelShuffle}(\mathrm{GELU}\left(\mathrm{Conv}3\times 3\left(F_{up1},C\to 4C\right)\right)),\\ {\hat{X}}_{2} & =\sigma \left(\mathrm{Conv}3\times 3(F_{up2},C\to 1)\right)\in R^{B\times 1\times 512\times 512} \end{array}$$

##### Segmentation Output Head

The segmentation head generates multi-frame segmentation masks with the same upsampling strategy.

(15) $$\begin{array}{cc} F_{up1} & =\mathrm{PixelShuffle}(\mathrm{Conv}3x3\left(F_{seg},C\to 4C\right)),\\ F_{up2} & =\mathrm{PixelShuffle}(\mathrm{GELU}\left(\mathrm{Conv}3x3\left(F_{up1},C\to 4C\right)\right)),\\ \hat{Y} & =\sigma \left(\mathrm{Conv}3\times 3(F_{up2},C\to 3)\right),\\ {\hat{Y}}_{1},{\hat{Y}}_{2},{\hat{Y}}_{3} & =\mathrm{Split}(\hat{Y},\dim=1) \end{array}$$

where $F_{up2}\in R^{B\times 96\times 512\times 512}$ and $\hat{Y}\in R^{B\times 3\times 512\times 512}$.
**Algorithm 1** TASC-SwinMT Forward Propagation**Require:** Input frames $X_{1},X_{3}\in R^{B\times 1\times 512\times 512}$**Ensure:** Interpolated frame ${\hat{X}}_{2}$, segmentation masks ${\hat{Y}}_{1},{\hat{Y}}_{2},{\hat{Y}}_{3}$1:Concatenate input frames: $X=\mathrm{Concat}\left(X_{1},X_{3}\right)\in R^{B\times 2\times 512\times 512}$2:Patch embedding: $F_{0}=\mathrm{PatchEmbed}\left(X\right)\in R^{B\times 96\times 128\times 128}$3:Extract shared features and record skip connections $S_{1},S_{2},S_{3},S_{4}$:4:**for** i=1 **to** 4 **do**5:    $F_{i}=\mathrm{SwinBlock}\left(F_{i-1}\right)$6:    $F_{i}=\mathrm{TALA}\left(F_{i}\right)$7:    $S_{i}=F_{i}$8:    **if** i<4 **then**9:        $F_{i}=\mathrm{PatchMerging}\left(F_{i}\right)$10:  **end if**11:**end for**12:Initialize dual decoders:13:$F_{\mathrm{interp}}=F_{4}$14:$F_{\mathrm{seg}}=F_{4}$15:**for** i=1 **to** 3 **do**16:    $F_{\mathrm{interp}}=\mathrm{PatchExpand}\left(F_{\mathrm{interp}}\right)$17:    $F_{\mathrm{interp}}=\mathrm{MSTAF}\left(F_{\mathrm{interp}},S_{4-i}\right)$18:    $F_{\mathrm{interp}}=\mathrm{SwinBlock}\left(F_{\mathrm{interp}}\right)$19:    $F_{\mathrm{seg}}=\mathrm{PatchExpand}\left(F_{\mathrm{seg}}\right)$20:    $F_{\mathrm{seg}}=\mathrm{MSTAF}\left(F_{\mathrm{seg}},S_{4-i}\right)$21:    $F_{\mathrm{seg}}=\mathrm{SwinBlock}\left(F_{\mathrm{seg}}\right)$22:    $F_{\mathrm{interp}},F_{\mathrm{seg}}=\mathrm{CTCI}\left(F_{\mathrm{interp}},F_{\mathrm{seg}}\right)$23:**end for**24:Generate final outputs: ${\hat{X}}_{2}=\mathrm{InterpHead}\left(F_{\mathrm{interp}}\right)$, $\hat{Y}=\mathrm{SegHead}\left(F_{\mathrm{seg}}\right)$25:Split multi-frame segmentation masks: ${\hat{Y}}_{1},{\hat{Y}}_{2},{\hat{Y}}_{3}=\mathrm{Split}\left(\hat{Y},\dim=1\right)$26:**return** ${\hat{X}}_{2},{\hat{Y}}_{1},{\hat{Y}}_{2},{\hat{Y}}_{3}$

### 3.3. Training and Evaluation Setup

#### 3.3.1. Loss Functions

This study constructs a dynamic multi-task loss function with fully learnable task weights to realize balanced joint optimization for interpolation and segmentation within the TASC-SwinMT framework. The overall loss formulation integrates weighted task loss terms and regularization constraints to avoid performance bias caused by single-task over-optimization in the training process. The complete mathematical expression of the total loss is defined as

(16) $$L_{\mathrm{total}}=\exp\left(-\theta_{1}\right)L_{\mathrm{interp}}+\exp\left(-\theta_{2}\right)L_{\mathrm{seg}}+\left(\theta_{1}+\theta_{2}\right)$$

##### Hybrid Interpolation Loss

The interpolation task adopts a hybrid loss combining L1 loss and Mean Squared Error (MSE) loss to retain fine anatomical details and maintain global structural smoothness. The mathematical definition is formulated as

(17) $$\begin{array}{cc} L_{L1} & =L1\mathrm{Loss}({\hat{X}}_{2},X_{2})\\ L_{\mathrm{MSE}} & =\mathrm{MSELoss}({\hat{X}}_{2},X_{2})\\ L_{\mathrm{interp}} & =\alpha \cdot L_{L1}+\left(1-\alpha \right)\cdot L_{\mathrm{MSE}} \end{array}$$

The hyperparameter α is set to 0.4 throughout all experiments. The hybrid loss inherits the detail preservation advantage of L1 loss and the structural smoothness characteristic of MSE loss to adapt to the reconstruction requirement of medical tomographic images.

##### Segmentation Loss

Binary cross-entropy loss is adopted to supervise the pixel-level segmentation prediction of lung tumor and left atrium regions. The loss function is expressed as

(18) $$L_{\mathrm{seg}}=\mathrm{BCELoss}\left({\hat{Y}}_{i},Y_{i}\right)$$

Benefiting from inherent structural design of the proposed framework, single Binary Cross-Entropy (BCE) loss can effectively complete segmentation supervision even under natural foreground–background imbalance of small lesions. Multi-scale spatial extraction and frequency-domain enhancement modules strengthen fine feature representation of tiny lesion areas and avoid background feature dominance. Joint optimization with interpolation task provides continuous spatiotemporal context to constrain model attention on sparse small targets. Adaptive weight adjustment of overall dynamic loss further offsets training bias caused by uneven pixel distribution. Discarding extra Dice Coefficient (Dice) loss avoids gradient interference from mixed loss combination and keeps model optimization steady. The optimization objective constrains the network to generate segmentation masks with accurate lesion boundary delineation and consistent anatomical morphology.

##### Mathematical Derivation of Learnable Weight Adaptation

Two independent trainable parameter vectors $\theta_{1}$ and $\theta_{2}$ are initialized as zero vectors at the start of model training. The normalized task weights for interpolation and segmentation are derived through exponential mapping:

(19) $$\begin{array}{cc} \theta_{1},\theta_{2} & =\mathrm{Parameter}(\mathrm{zeros}(2))\\ w_{1} & =\exp\left(-\theta_{1}\right),\;w_{2}=\exp\left(-\theta_{2}\right) \end{array}$$

The exponential mapping constrains task weights within a positive bounded interval and prevents extreme weight values from disrupting training stability. The regularization term $\left(\theta_{1}+\theta_{2}\right)$ is introduced to constrain the magnitude of learnable parameters and avoid overfitting caused by excessive parameter deviation.

During backpropagation, the gradient of total loss with respect to learnable parameters is calculated as:

(20) $$\begin{array}{cc} \frac{\partial L_{\mathrm{total}}}{\partial \theta_{1}} & =-w_{1}L_{\mathrm{interp}}+1,\\ \frac{\partial L_{\mathrm{total}}}{\partial \theta_{2}} & =-w_{2}L_{\mathrm{seg}}+1. \end{array}$$

The optimizer updates $\theta_{1}$ and $\theta_{2}$ iteratively according to the calculated gradient. The corresponding task weights $w_{1}$ and $w_{2}$ adjust dynamically with the changing loss magnitude of two tasks. The weighting mechanism automatically suppresses the optimization dominance of simple tasks and strengthens the constraint of hard tasks to realize adaptive gradient equilibrium.

All learnable weight parameters participate in model training together with Swin Transformer backbone, TALA, MSTAF and CTCI module parameters. The whole optimization process follows an end-to-end joint learning paradigm without additional independent optimization steps or separate loss tuning pipelines. The adaptive weight updating relies entirely on the global backpropagation of the multi-task loss to guarantee the inherent consistency of feature learning and loss balancing.

#### 3.3.2. Training Configuration

To ensure the reproducibility of experimental results, we adopted a unified training configuration for both datasets:

1. Optimizer: Adam optimizer with dynamically learnable learning rate and weight decay, initialized as $1\times 10^{-4}$ and $1\times 10^{-5}$, respectively, and both parameters are automatically optimized during training;
2. Batch size: 2 (constrained by GPU memory);
3. Training epochs: 100 epochs with early stopping (patience = 10);
4. Training stability measures: Mixed precision training (FP16) and gradient clipping;
5. Parameter initialization: He normal initialization for convolutional layers.

All key notations used in this work are defined in Table 3.

## 4. Experiments

This section details the experimental setup in terms of hardware and software, and presents quantitative, qualitative, and ablation results on the MSD Task02_Heart and Task06_Lung datasets, together with expanded baseline comparisons.

### 4.1. Evaluation Metrics

To evaluate the performance of the proposed TASC-SwinMT model on interpolation and segmentation tasks, we adopt a set of widely recognized quantitative metrics in medical image analysis, including both pixel-level reconstruction metrics for interpolation and region-level overlap metrics for segmentation. The detailed definitions and mathematical formulations of these metrics are as follows:

#### 4.1.1. Interpolation Evaluation Metrics

##### Peak Signal-to-Noise Ratio (PSNR)

PSNR measures the pixel-level reconstruction quality of interpolated CT frames. It is calculated based on the mean squared error (MSE) between the predicted interpolated frame ${\hat{X}}_{2}$ and the ground truth frame $X_{2}$:

(21) $$\begin{array}{cc} \mathrm{MSE} & =\frac{1}{H\times W}\sum_{h=1}^{H}\sum_{w=1}^{W}{\left({\hat{X}}_{2}\left(h,w\right)-X_{2}\left(h,w\right)\right)}^{2}\\ \mathrm{PSNR} & =10\times \log_{10}\left(\frac{\max{\left(X_{2}\right)}^{2}}{\mathrm{MSE}}\right) \end{array}$$

where *H* and *W* denote the height and width of the CT frame, and $\max(X_{2})$ is the maximum pixel value of the ground truth frame (normalized to 1 in our experiments).

##### Structural Similarity Index (SSIM)

SSIM evaluates the overall structural consistency between the predicted interpolated frame and the ground truth frame from three dimensions including luminance, contrast, and structural similarity. The metric value ranges from 0 to 1, where a higher value indicates better structural reconstruction quality.

(22) $$\begin{array}{cc} A_{\mathrm{SSIM}} & =\left(2\mu_{X_{2}}\mu_{{\hat{X}}_{2}}+C_{1}\right)\left(2\sigma_{X_{2}{\hat{X}}_{2}}+C_{2}\right)\\ B_{\mathrm{SSIM}} & =\left(\mu_{X_{2}}^{2}+\mu_{{\hat{X}}_{2}}^{2}+C_{1}\right)\left(\sigma_{X_{2}}^{2}+\sigma_{{\hat{X}}_{2}}^{2}+C_{2}\right)\\ \mathrm{SSIM}(X_{2},{\hat{X}}_{2}) & =\frac{A_{\mathrm{SSIM}}}{B_{\mathrm{SSIM}}} \end{array}$$

where $\mu_{X_{2}}$ and $\mu_{{\hat{X}}_{2}}$ are the mean pixel values of $X_{2}$ and ${\hat{X}}_{2}$, $\sigma_{X_{2}}^{2}$ and $\sigma_{{\hat{X}}_{2}}^{2}$ are the corresponding pixel variances, $\sigma_{X_{2}{\hat{X}}_{2}}$ denotes the covariance between the two frames, and $C_{1}={\left(0.01\times \max\left(X_{2}\right)\right)}^{2}$ and $C_{2}={\left(0.03\times \max\left(X_{2}\right)\right)}^{2}$ are constant regularization terms to avoid denominator being zero.

##### Edge Structural Similarity Index (EdgeSSIM)

EdgeSSIM is a dedicated variant of SSIM that only focuses on the structural similarity of anatomical edge regions. It calculates the average SSIM value of pixels located on anatomical contours, which can more accurately reflect the reconstruction fidelity of lesion and tissue boundaries.

(23) $$\begin{array}{cc} S_{\mathrm{edge}} & =\sum_{\left(h,w\right)\in {\mathrm{edge}}_{\mathrm{mask}}}\mathrm{SSIM}\left(X_{2}\left(h,w\right),{\hat{X}}_{2}\left(h,w\right)\right)\\ \mathrm{EdgeSSIM} & =\frac{S_{\mathrm{edge}}}{N_{\mathrm{edge}}} \end{array}$$

where ${\mathrm{edge}}_{\mathrm{mask}}$ represents the anatomical edge mask extracted by the Sobel operator, and $N_{\mathrm{edge}}$ is the total number of edge pixels contained in the mask.

##### Learned Perceptual Image Patch Similarity (LPIPS)

LPIPS quantifies the perceptual similarity between frames using a pre-trained deep network:

(24) $$\begin{array}{cc} \Delta_{l,h,w,c} & ={∥\frac{\phi_{l}{\left(X_{2}\right)}_{h,w,c}}{∥\phi_{l}{\left(X_{2}\right)}_{h,w,c}{∥}_{2}}-\frac{\phi_{l}{\left({\hat{X}}_{2}\right)}_{h,w,c}}{∥\phi_{l}{\left({\hat{X}}_{2}\right)}_{h,w,c}{∥}_{2}}∥}_{2}^{2}\\ D_{l} & =\frac{1}{H_{l}W_{l}C_{l}}\sum_{h=1}^{H_{l}}\sum_{w=1}^{W_{l}}\sum_{c=1}^{C_{l}}\Delta_{l,h,w,c}\\ \mathrm{LPIPS}(X_{2},{\hat{X}}_{2}) & =\frac{1}{L}\sum_{l=1}^{L}D_{l} \end{array}$$

where $\phi_{l}$ denotes the feature map of the *l*-th layer of the pre-trained VGG network, and $H_{l},W_{l},C_{l}$ are the height, width, and channel of the *l*-th layer feature map.

#### 4.1.2. Segmentation Evaluation Metrics

##### Dice Coefficient

Dice coefficient measures the overlap between the predicted segmentation mask ${\hat{Y}}_{i}$ and the ground truth mask $Y_{i}$ (for i=1,2,3), with values ranging from 0 to 1:

(25) $$\begin{array}{cc} I_{i} & =\sum_{h=1}^{H}\sum_{w=1}^{W}\left({\hat{Y}}_{i}\left(h,w\right)\times Y_{i}\left(h,w\right)\right)\\ P_{i} & =\sum_{h=1}^{H}\sum_{w=1}^{W}{\hat{Y}}_{i}{\left(h,w\right)}^{2},\;G_{i}=\sum_{h=1}^{H}\sum_{w=1}^{W}Y_{i}{\left(h,w\right)}^{2}\\ \mathrm{Dice}({\hat{Y}}_{i},Y_{i}) & =\frac{2\times |{\hat{Y}}_{i}\cap Y_{i}|}{|{\hat{Y}}_{i}\left|+\right|Y_{i}|}=\frac{2I_{i}}{P_{i}+G_{i}} \end{array}$$

##### Intersection over Union (IoU)

IoU (Jaccard index) is a complementary metric to Dice, calculating the ratio of the intersection to the union of predicted and ground truth masks:

(26) $$\begin{array}{cc} U_{i} & =P_{i}+G_{i}-I_{i}\\ \mathrm{IoU}({\hat{Y}}_{i},Y_{i}) & =\frac{|{\hat{Y}}_{i}\cap Y_{i}|}{|{\hat{Y}}_{i}\cup Y_{i}|}=\frac{I_{i}}{U_{i}} \end{array}$$

##### Precision and Recall

Precision evaluates the accuracy of positive predictions, while recall measures the completeness of lesion detection:

(27) $$\mathrm{Precision}=\frac{|{\hat{Y}}_{i}\cap Y_{i}|}{|{\hat{Y}}_{i}|},\;\mathrm{Recall}=\frac{|{\hat{Y}}_{i}\cap Y_{i}|}{|Y_{i}|}$$

##### Temporal Consistency Score (TCS)

TCS quantifies the temporal smoothness of segmentation masks across consecutive frames:

(28) $$\begin{array}{cc} M_{h,w} & =\frac{{\hat{Y}}_{1}\left(h,w\right)+{\hat{Y}}_{3}\left(h,w\right)}{2}\\ E_{h,w} & =\left|{\hat{Y}}_{2}\left(h,w\right)-M_{h,w}\right|\\ \mathrm{TCS} & =\frac{1}{H\times W}\sum_{h=1}^{H}\sum_{w=1}^{W}\left(1-E_{h,w}\right) \end{array}$$

### 4.2. Experimental Setup

#### 4.2.1. Hardware/Software Environment

1. Hardware: Intel Core i9-12900K (16 cores/32 threads), 64 GB DDR4 RAM, NVIDIA RTX 4090 (24 GB VRAM), 2TB NVMe SSD.
2. Software: Ubuntu 20.04 LTS, Python 3.9.18, PyTorch 2.0.1, CUDA 11.7, cuDNN 8.5.0, nibabel 5.1.0, scikit-image 0.21.0, numpy 1.26.0, matplotlib 3.8.0.

Statistical significance was assessed using Welch’s *t*-test across five independent experiments, where *, **, and *** represent p<0.05, p<0.01, and p<0.001, respectively, compared with our proposed method, and the best performance in each metric column is marked in bold.

#### 4.2.2. Expanded Baseline and Ablated Models

We expand baseline models (covering CNN/Transformer-based single-task methods) and ablated variants (multi-dimensional ablation):

##### Baseline Models

1. SwinUNet-Interp: SwinUNet for interpolation [26].
2. TransUNet-Interp: TransUNet for interpolation [64].
3. VTN-Interp: Transformer-based multi-scale interpolation model [16].
4. nnU-Net-Seg: nnU-Net for segmentation [22].
5. UNet++-Seg: U-Net++ for segmentation [23].
6. Swin UNETR-Seg: Swin UNETR for segmentation [11].

##### Ablated Variants (Proposed Model)

1. IndEnc: Baseline configuration adopting independent encoders for interpolation and segmentation tasks, without shared feature learning.
2. +TALA: Base framework embedded only with the Task-Aware Lightweight Adapter module.
3. +CTCI: Base framework embedded only with the Cross-Task Collaborative Interaction module.
4. +MSTAF: Base framework embedded only with the Multi-Scale Task Alignment Fusion module.
5. +TALA+CTCI: Base framework integrated with both TALA and CTCI modules for pairwise component validation.
6. +TALA+MSTAF: Base framework integrated with both TALA and MSTAF modules for pairwise component validation.
7. +CTCI+MSTAF: Base framework integrated with both CTCI and MSTAF modules for pairwise component validation.
8. StaticLoss: Full framework replacing learnable dynamic weights with fixed weighted multi-task loss.
9. Loss0.3: Full framework adopting hybrid interpolation loss with balance parameter set to α=0.3.
10. Loss0.5: Full framework adopting hybrid interpolation loss with balance parameter set to α=0.5.

**Figure 5:**
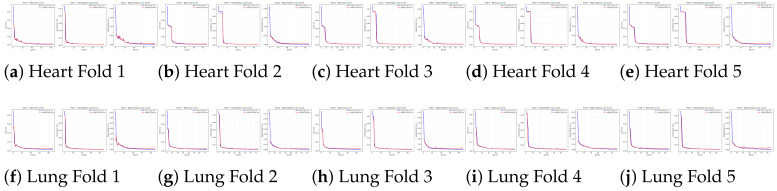
Training and validation loss curves of the proposed TASC-SwinMT model under five-fold cross-validation.

To ensure a fair comparison between different models, all models are trained with the same configuration, including batch size, number of training epochs, and optimizer parameters. To reduce the randomness of experimental results, all quantitative results are reported as the mean ± standard deviation over five independent repeated experiments. The training and validation loss curves under five-fold cross-validation are visualized in Figure 5.

### 4.3. Baseline Comparison

This subsection provides quantitative comparison results among the proposed TASC-SwinMT and mainstream baseline networks for image interpolation and medical image segmentation. All baseline models follow identical training configurations to guarantee fair experimental comparison. The proposed TASC-SwinMT obtains the optimal values on all core metrics and delivers comprehensive performance superior to all compared baseline methods.

#### 4.3.1. Baseline Comparison Results on Heart Dataset (MSD Task02_Heart)

Table 4 and Table 5 present the quantitative results of interpolation and segmentation tasks on the Heart dataset.

**Figure 6:**
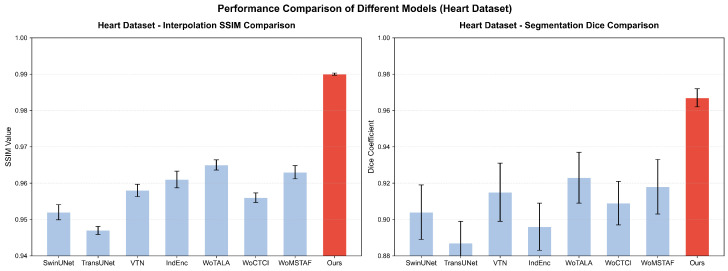
Heart dataset: SSIM (interpolation) and Dice (segmentation).

**Figure 7:**
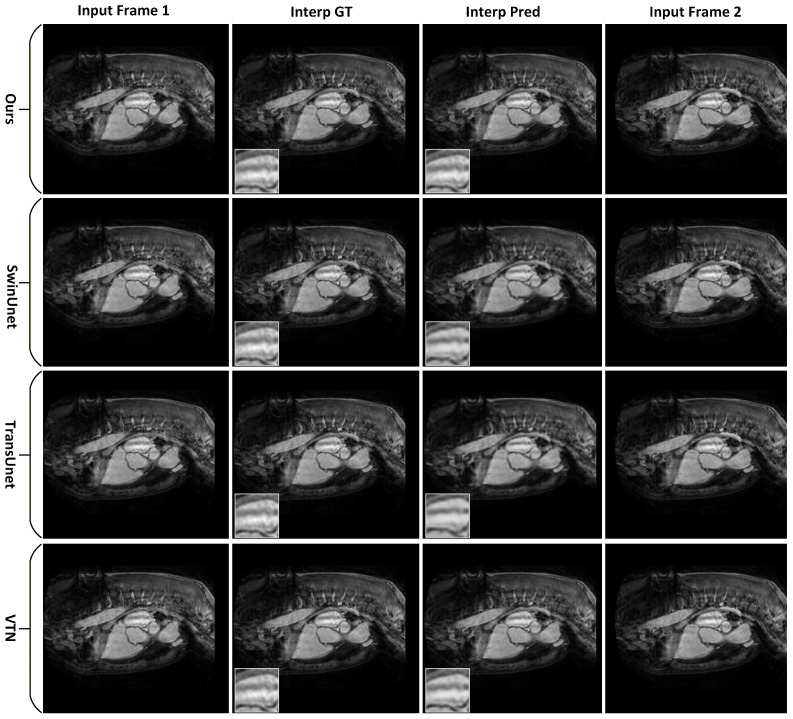
Heart Dataset: Interpolation Result Visualization.

**Figure 8:**
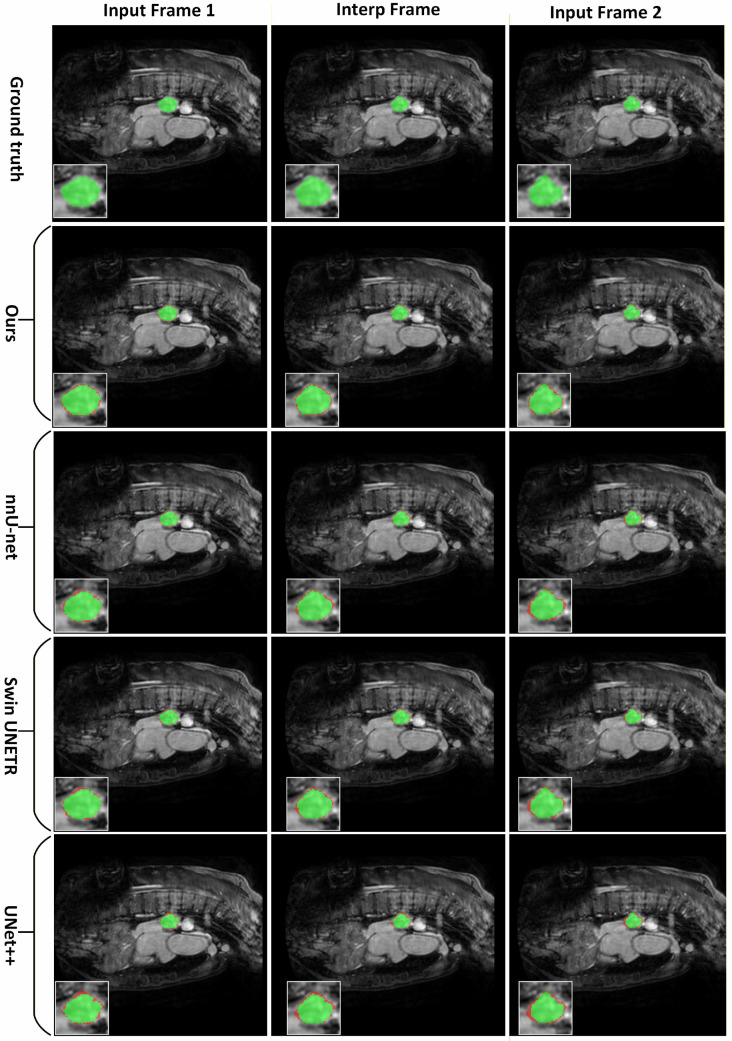
Heart dataset: segmentation result visualization.

Figure 6, Figure 7 and Figure 8 show quantitative and qualitative results on the Heart dataset.

#### 4.3.2. Baseline Comparison Results on Lung Dataset (MSD Task06_Lung)

Table 6 and Table 7 present results on the Lung dataset.

**Figure 9:**
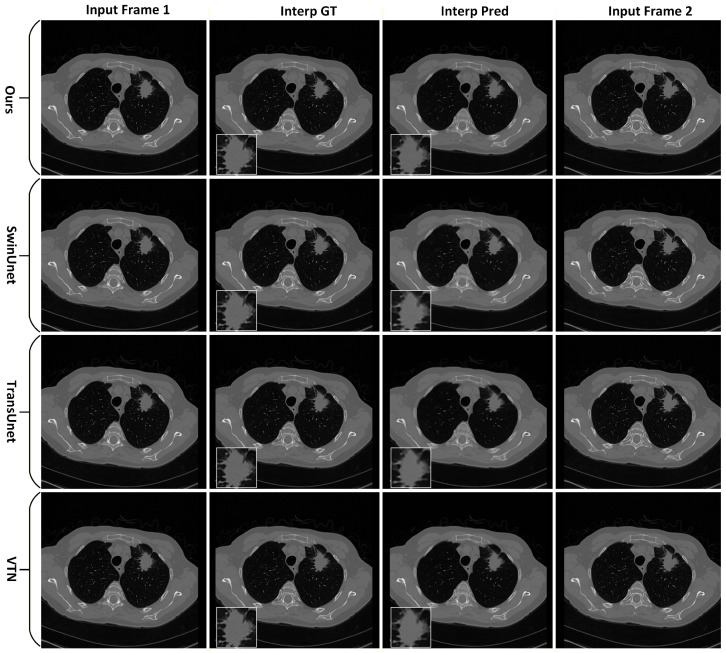
Lung dataset: interpolation result visualization.

**Figure 10:**
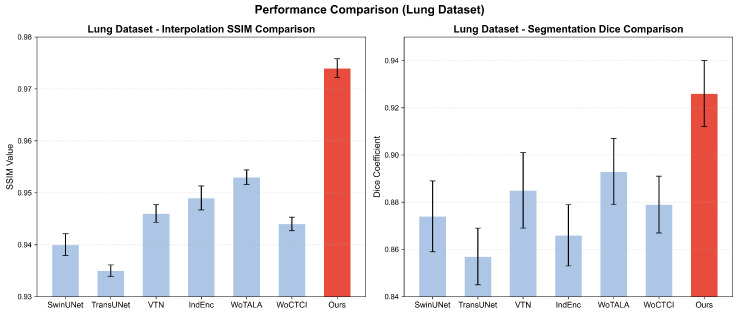
Lung dataset: SSIM (interpolation) and Dice (segmentation).

**Figure 11:**
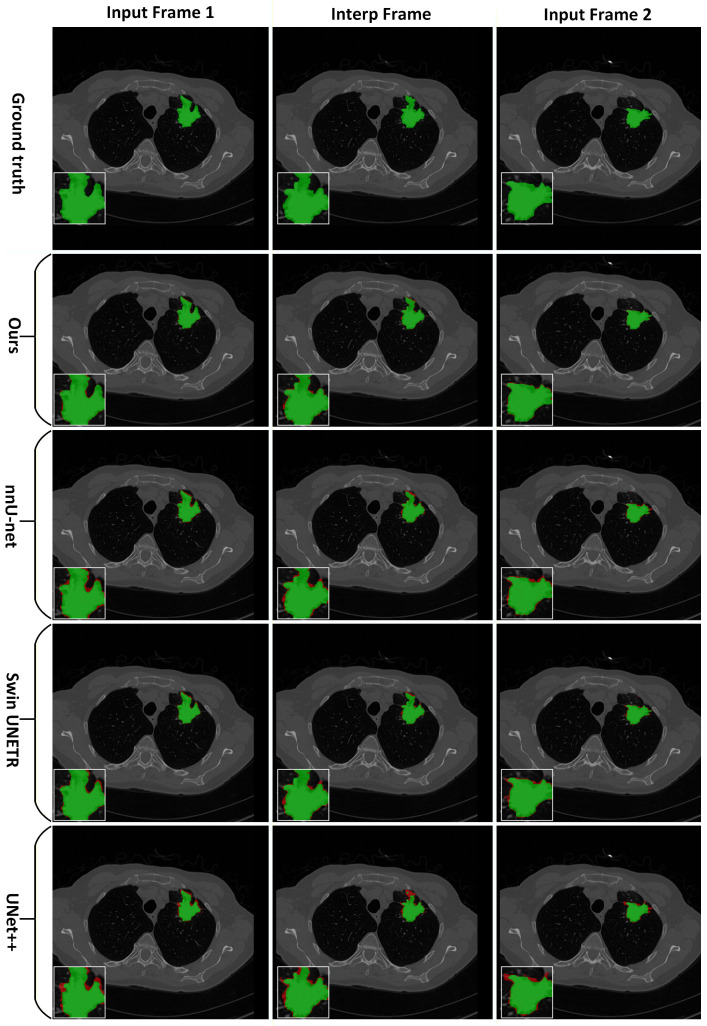
Lung dataset: segmentation result visualization.

Figure 9, Figure 10 and Figure 11 show quantitative and qualitative results on the Lung dataset.

### 4.4. Ablation Studies

This subsection conducts comprehensive ablation experiments to verify the effectiveness of each core component and module interaction effect of the proposed framework. All ablation variants are tested under identical training and evaluation configurations to ensure fair performance comparison.

#### 4.4.1. Model Variant Ablation

PSNR and SSIM quantify interpolation reconstruction quality while Dice and Recall reflect segmentation accuracy. We design systematic ablation schemes covering standalone module embedding and pairwise module combination embedding, which can fully reveal the individual contribution and interactive synergy of each component. The ablation results of variant modules on the Heart and Lung dataset are presented in Table 8 and Table 9.

#### 4.4.2. Loss Weight Ablation

Table 10 presents the influence of multi-task loss weight configuration on core PSNR, Dice and total loss model performance.

#### 4.4.3. Core Component Contribution Analysis

Table 11 and Table 12 quantifies the performance contribution of each core component.

The independent encoder baseline IndEnc obtains the lowest performance on all metrics across two datasets. The embedding of any single module brings stable performance improvement over IndEnc. On the heart dataset, adding TALA raises PSNR by 1.13 dB and Dice by 2.30 percentage points, while adding MSTAF increases PSNR by 1.35 dB and Dice by 2.90 percentage points. Pairwise module combinations further outperform any single module with evident synergistic effects. The combination of TALA and MSTAF achieves 40.61 dB PSNR on the heart dataset, which exceeds the standalone TALA by 1.15 dB and standalone MSTAF by 0.93 dB. All pairwise combinations maintain lower metric values than the full framework with all three modules integrated.

The same performance trend can be observed on the lung dataset. Single module embedding brings consistent gain over IndEnc, and pairwise combinations produce further performance promotion without reaching the optimal level of the complete model. The layered performance distribution from IndEnc to single module, pairwise combination and full framework validates the rationality of module design and the positive interaction effect between different components.

### 4.5. Comparative Experiments of Multi-Task Learning Methods

To further validate the superiority of the proposed TASC-SwinMT multi-task framework, four representative mainstream multi-task learning methods are selected for comprehensive comparison, including direct feature concatenation, Shared-Bottom hard parameter sharing, cross-task feature concatenation, and Mixture of Experts (MoE). This subsection first introduces the basic principle of each method, and derives the mathematical formulation of each method following the symbol definition system in the Methods section.

Direct Feature Concatenation

Direct feature concatenation superposes interpolation and segmentation features along the channel dimension without adaptive feature calibration and cross-task interactive optimization. The mathematical formulation is defined as:

(29) $$F_{cat}=\mathrm{Concat}\left(F_{interp},F_{seg},\dim=1\right)$$

This method only implements simple feature aggregation and ignores the inherent feature distribution discrepancy and semantic mismatch between heterogeneous tasks [65].

Shared-Bottom

Shared-Bottom adopts a globally shared encoder to extract universal low-level spatial features, and configures independent task-specific prediction heads for interpolation and segmentation, respectively. The mathematical formulation is defined as:

(30) $$F_{share}={\mathrm{Encoder}}_{share}\left(X\right),\;{\hat{X}}_{2}={\mathrm{Head}}_{interp}\left(F_{share}\right),\;{\hat{Y}}_{i}={\mathrm{Head}}_{seg}\left(F_{share}\right)$$

The shared underlying structure reduces computational redundancy, and independent task branches maintain basic task-specific learning capability [66].

Cross-Task Feature Concatenation

Cross-task feature concatenation performs layer-wise feature delivery between two task branches and fuses multi-scale intermediate features at different network stages. The mathematical formulation is defined as:

(31) $$F_{cross}=\mathrm{Concat}\left(F_{interp}^{l},F_{seg}^{l},\dim=1\right),\;l=1,2,3,4$$

where *l* denotes the index of multi-scale feature extraction layers. This method captures shallow cross-task correlations but lacks deep feature alignment and dynamic gating regulation [67].

Mixture of Experts (MoE)

MoE constructs multiple expert sub-networks for diversified feature representation, and integrates the output of each expert through task-agnostic global gating weights. The mathematical formulation is defined as:

(32) $$F_{moe}=\sum_{k=1}^{K}g{\left(x\right)}_{k}\cdot {\mathrm{Expert}}_{k}\left(X\right)$$

where *K* represents the total number of expert networks, and $g{\left(x\right)}_{k}$ denotes the normalized gating weight corresponding to the *k*-th expert. MoE enriches feature diversity via multi-expert ensemble but cannot achieve task-adaptive feature modulation [68].

All comparison methods adopt the identical Swin Transformer backbone and training configuration as the proposed TASC-SwinMT to ensure fair experimental validation. Quantitative interpolation and segmentation results on cardiac MRI and lung CT datasets are reported in Table 13 and Table 14.

Quantitative results reveal obvious performance discrepancies across all compared multi-task methods. In terms of interpolation metrics, Direct Concatenation only achieves 35.42 dB PSNR and 0.936 SSIM, while its segmentation Dice is merely 0.871, with all indexes lower than the original single-task baselines. Shared-Bottom gains limited promotion and reaches 36.35 dB PSNR, 0.945 SSIM and 0.883 Dice, keeping a numerical level close to but different from baseline results. Cross-Task Concatenation further improves to 37.68 dB PSNR, 0.954 SSIM and 0.896 Dice, and slightly exceeds single-task baselines on all metrics. MoE performs best among the four comparison methods with 37.92 dB PSNR, 0.957 SSIM and 0.901 Dice. The proposed TASC-SwinMT obtains 41.50 dB PSNR and 0.967 Dice, which surpasses MoE by 3.58 dB in PSNR and 6.6 percentage points in Dice coefficient. Such significant quantitative gaps fully demonstrate the outstanding superiority of the cross-task collaborative mechanism embedded in our framework.

### 4.6. Multi-Task Balancing Strategy Evaluation and Training Dynamics Analysis

In multi-task medical image learning, heterogeneous optimization objectives of image interpolation and segmentation easily cause training bias and performance imbalance with fixed weight loss configuration. To address this issue, three typical multi-task loss balancing strategies including fixed weighted loss, GradNorm gradient normalization (GradNorm) [69] and dynamic weight average (DWA) [70] are comprehensively evaluated and compared with the proposed learnable dynamic loss strategy in this section. The mathematical formulations and core characteristics of three mainstream balancing strategies are defined as follows.

Fixed weighted loss adopts manually preset constant weights to superpose task loss terms:

$$L_{\mathrm{fix}}=w_{1}L_{\mathrm{interp}}+w_{2}L_{\mathrm{seg}}$$

where $w_{1}$ and $w_{2}$ are fixed hyperparameters, which fail to adaptively adjust with the dynamic change of task optimization difficulty during training.

GradNorm realizes task balance by normalizing gradient magnitude of different tasks

$$L_{\mathrm{grad}}=\lambda_{1}L_{\mathrm{interp}}+\lambda_{2}L_{\mathrm{seg}},\;\left\{\lambda_{1},\lambda_{2}\right\}\leftarrow \mathrm{GradNorm}\left(\nabla L_{\mathrm{interp}},\nabla L_{\mathrm{seg}}\right)$$

It adaptively updates task weights by constraining gradient amplitude consistency, yet ignores the intrinsic feature correlation between interpolation and segmentation tasks.

DWA assigns dynamic weights according to the decay rate of task loss:

$$L_{\mathrm{dwa}}=\lambda_{1}L_{\mathrm{interp}}+\lambda_{2}L_{\mathrm{seg}},\;\lambda_{i}\propto \exp\left(-r_{i}/\tau \right)$$

where $r_{i}$ denotes the loss decline rate of each task and τ is the temperature coefficient, which relies only on loss statistics and lacks explicit constraint on task optimization equilibrium.

Different from the above strategies, the proposed learnable dynamic multi-task loss introduces trainable parameters with exponential mapping and regularization constraint:

$$L_{\mathrm{total}}=\exp\left(-\theta_{1}\right)L_{\mathrm{interp}}+\exp\left(-\theta_{2}\right)L_{\mathrm{seg}}+\left(\theta_{1}+\theta_{2}\right)$$

This design enables end-to-end adaptive weight optimization for interpolation and segmentation, effectively balancing heterogeneous objectives and avoiding single-task dominance in joint training.

The experimental results displayed in Table 15 show distinct performance preferences among the three strategies on Cardiac Dataset, and Grad-Norm and DWA exhibit extremely similar effects in all evaluation metrics. DWA and Grad-Norm achieve slightly higher PSNR and SSIM values on the interpolation task, which is attributed to their gradient-based weight adjustment mechanism that prioritizes the optimization of the interpolation task with lower learning difficulty. In contrast, the proposed dynamic loss strategy achieves a significant performance improvement on the segmentation task, with Dice coefficient reaching 0.967 and Recall reaching 0.968, which far exceed the 0.918 Dice of Grad-Norm and 0.932 Dice of DWA. This result verifies that the proposed method effectively balances the optimization of heterogeneous dual tasks, and significantly enhances the segmentation performance that is more critical for clinical diagnosis, while maintaining stable interpolation reconstruction quality.

The training dynamics of the three multi-task balancing strategies are further analyzed on cardiac MRI data to reveal the convergence behavior and collaborative learning mechanism between the interpolation and segmentation tasks. This analysis validates the stability of joint optimization and the effectiveness of cross-task feature interaction during model training for different strategies.

**Figure 12:**
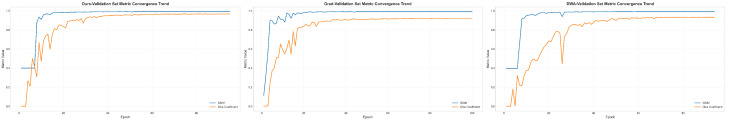
Validation metric convergence trend of three multi-task balancing strategies.

The convergence trend of core validation metrics for the three strategies is illustrated in Figure 12, which contains three subgraphs corresponding to the proposed dynamic loss, Grad-Norm, and DWA, respectively. The three strategies exhibit significantly different convergence characteristics across 100 training epochs with clear differences at key training nodes.

For the proposed dynamic loss strategy, the interpolation SSIM remains at 0.4000 in the first 7 epochs, rises rapidly to 0.8779 at the 8th epoch, and enters a steady growth phase. At the 15th epoch, the SSIM reaches 0.9779, and at the 25th epoch, it stabilizes above 0.9856. At the 50th epoch, the SSIM reaches 0.9887, and at the 95th epoch, it reaches 0.9907 with a standard deviation of only 0.0006 in the final 50 epochs. The segmentation Dice coefficient starts from 0.0000 in the first 3 epochs, rises to 0.2676 at the 4th epoch, reaches 0.6668 at the 9th epoch, and climbs to 0.8935 at the 25th epoch. It continues to grow steadily to 0.9554 at the 50th epoch, and stabilizes at 0.9657 at the 95th epoch with controlled fluctuations throughout the training process.

For Grad-Norm, the interpolation SSIM starts at 0.1113 at the 1st epoch, surges rapidly to 0.8972 at the 4th epoch, and climbs to 0.9449 at the 8th epoch, showing fast early-stage convergence. It maintains stable growth afterward, reaching 0.9851 at the 25th epoch and 0.9910 at the 100th epoch. For DWA, the interpolation SSIM stays at 0.3970 in the first 6 epochs, jumps to 0.6538 at the 7th epoch, and rises to 0.9135 at the 8th epoch, with a slower startup speed than Grad-Norm but steady growth to 0.9857 at the 25th epoch and 0.9907 at the 94th epoch. Both strategies maintain high interpolation SSIM in the late training stage, but their segmentation Dice coefficients show distinct slow growth and low final values. Grad-Norm’s segmentation Dice reaches 0.6522 at the 9th epoch, fluctuates mildly, and only hits 0.8789 at the 25th epoch, stabilizing at 0.9110 at the 50th epoch and 0.9200 at the 100th epoch with no late-stage improvement. DWA’s segmentation Dice grows more slowly, reaching 0.3168 at the 9th epoch and 0.7678 at the 25th epoch, stabilizing at 0.8993 at the 50th epoch and 0.9320 at the 94th epoch, still far below the proposed method.

The long-term imbalance between the two metrics reflects that Grad-Norm and DWA cannot effectively balance the optimization of dual tasks, and the model learning is consistently biased towards the interpolation task. The overall synchronous upward trend of the two metrics for the proposed method indicates that the framework achieves balanced learning for both tasks without long-term performance suppression on either task.

**Figure 13:**
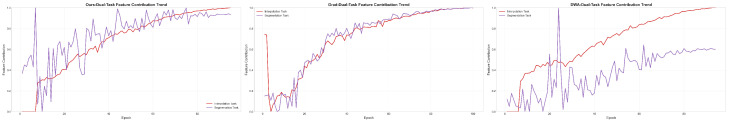
Dual-task feature contribution trend of three multi-task balancing strategies.

The dual-task feature contribution trend of the three strategies is visualized in Figure 13. The feature contribution patterns further reveal the underlying mechanism of the performance differences between the strategies.

For the proposed dynamic loss strategy, the interpolation feature contribution stays at 0.0000 in the first 7 epochs, which is highly consistent with the stagnant interpolation SSIM at 0.4000 during the same period. It rises rapidly to 0.2753 at the 8th epoch and reaches 0.3237 at the 15th epoch, synchronizing with the sharp growth and steady improvement of interpolation SSIM, and finally stabilizes at 1.0000 at the 95th epoch, matching the saturated reconstruction performance. The segmentation feature contribution maintains at 0.3682 to 0.5442 in the first 6 epochs, corresponding to the initial start-up of the segmentation Dice coefficient. It fluctuates moderately between 0.0742 and 0.8040 from the 9th to the 43th epoch, which is consistent with the continuous and stable growth of segmentation Dice without performance collapse, and ultimately stabilizes at 0.9363 at the 95th epoch. The synchronous evolution between feature contribution dynamics and metric growth trends confirms that the adaptive feature allocation adjustment between the dual tasks matches the real-time optimization requirements of the segmentation task.

For Grad-Norm, the interpolation feature contribution starts at 0.7453 at the 1st epoch and drops to 0.0000 at the 4th epoch, which directly corresponds to the rapid surge of interpolation SSIM in the early training stage. It stays within 0.1542 to 0.9986 throughout training and finally reaches 1.0000 at the 95th epoch, supporting the high and stable interpolation reconstruction performance. In sharp contrast, its segmentation feature contribution remains low at 0.0170 to 0.1807 in the first 14 epochs, which explains the slow growth and delayed convergence of segmentation Dice in the early and middle stages, and only fluctuates between 0.5292 and 0.9994 with small amplitude after the 20th epoch, failing to drive further improvement of segmentation performance. For DWA, the interpolation feature contribution remains near 0.0000 in the first 6 epochs, corresponding to the slow start-up of its interpolation SSIM, then rises to 0.2928 at the 7th epoch and grows steadily to 1.0000 at the 93rd epoch, showing a completely different feature evolution trajectory from Grad-Norm. Its segmentation feature contribution stays at a low level for most training stages, matching the unsatisfactory growth rate and final convergence value of segmentation Dice, and only stabilizes at 0.6016 at the 93rd epoch, far lower than Grad-Norm and the proposed method. This persistent imbalance between dual-task feature contribution and the mismatched correlation with segmentation metric growth indicates that Grad-Norm and DWA fail to guide the model to learn effective feature representations for the segmentation task, with most learning resources occupied by the interpolation task.

Despite the moderate oscillation in segmentation feature contribution, the proposed dynamic loss strategy achieves significantly higher final segmentation Dice and Recall values than Grad-Norm and DWA. The strong positive correlation between adaptive oscillatory feature allocation and continuous segmentation metric improvement demonstrates that this mechanism is more conducive to breaking the optimization bottleneck of the segmentation task. In contrast, the excessively stable but extremely imbalanced feature allocation of Grad-Norm and huge oscillation in DWA leads to a weak correlation with segmentation performance growth, which ultimately limits the upper bound of segmentation performance.

### 4.7. Single-Task vs. Multi-Task Comparison

A controlled experiment is conducted to compare the proposed multi-task model (Ours-MT) with its single-task counterparts, namely Ours-Interp for independent interpolation and Ours-Seg for independent segmentation. All models employ the same network backbone, training setup, and hyperparameters. The single-task variants retain the corresponding decoder and task-specific modules while removing cross-task interaction mechanisms and shared encoder optimization for the other task.

#### 4.7.1. Heart Dataset (MSD Task02_Heart)

Table 16 and Table 17 present the quantitative comparison results on the Heart dataset.

#### 4.7.2. Lung Dataset (MSD Task06_Lung)

We report the interpolation and segmentation performance separately on the Lung dataset, as shown in Table 18 and Table 19.

### 4.8. Interpretability Visualization Experiments

**Figure 14:**
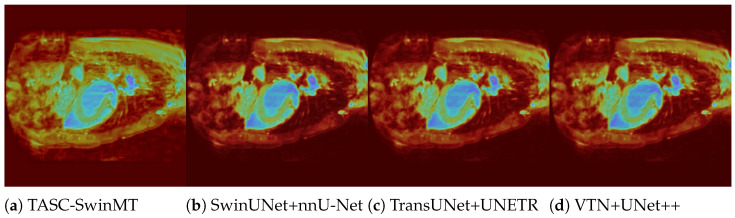
Interpretability visualization results on Heart dataset.

**Figure 15:**
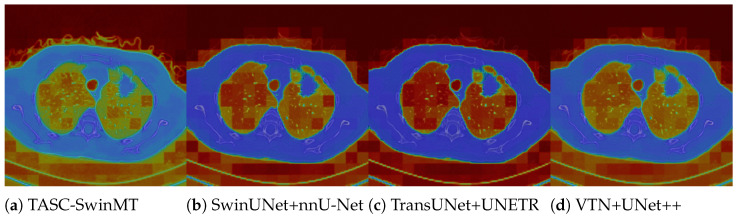
Interpretability visualization results on Lung dataset.

We conduct interpretability visualization experiments on both Lung and Heart datasets to analyze the feature learning mechanism of the model. We extract four representative feature maps for comprehensive visualization and comparison. These feature maps cover the cross-task collaborative attention map, the anatomical edge reconstruction feature, the frequency-domain global feature, and the segmentation boundary attention map. All feature maps are normalized to the range of zero to one. We adopt a hot color gradient from blue to red for clear visualization as is shown in Figure 14 and Figure 15.

## 5. Discussion

### 5.1. Result Analysis

Quantitative evaluations on two public medical datasets validate that the proposed TASC-SwinMT achieves superior performance in slice interpolation and lesion segmentation tasks. The multi-task collaborative modeling design outperforms conventional CNN and Transformer-based single-task baselines with significant statistical differences, demonstrating the necessity and rationality of joint learning for lung CT and cardiac MRI analysis. Quantitative metric distributions show that our method yields narrow error fluctuations and stable numerical repeatability across repeated experiments.

The constructed multi-task framework exhibits strong robustness against complex clinical imaging characteristics: it maintains reliable reconstruction and segmentation performance on cardiac MRI with stable structural preservation, as well as lung CT data with irregular tumor morphology and ambiguous tissue boundaries. Cross-dataset validation further verifies the satisfactory generalization capability of the proposed method for diverse thoracic anatomical structures. Qualitative visual samples consistently confirm stable output quality under variable imaging conditions, where lung CT interpolation preserves complete anatomical details consistent with ground truth, and segmentation accurately delineates lung tumor regions, validating the practical efficacy of the designed multi-task mechanism.

Ablation experiments verify the contribution and rationality of each core module and shared encoder design. Removing any key module or replacing the shared encoder with independent single-task encoders leads to obvious performance degradation. Excluding the MSTAF module induces feature distribution mismatch between encoder skip connections and decoder upsampling branches, deteriorating interpolation reconstruction and anatomical edge retention on both cardiac and pulmonary data, with more severe performance drops observed on the heart dataset. The embedded learnable dynamic multi-task loss function effectively balances optimization objectives between reconstruction and segmentation, achieving stable training equilibrium.

1. Each designed module targets inherent bottlenecks in multi-task feature learning and brings steady performance gains over the independent encoder baseline. The MSTAF module delivers the most prominent performance improvement by rectifying feature distribution mismatch via bidirectional cross-attention and frequency enhancement, stabilizing multi-scale feature fusion and achieving maximum increments in PSNR, SSIM and Dice. The TALA module further boosts performance by integrating spatial dual-bottleneck extraction and global frequency modeling, realizing task-oriented feature modulation with lightweight parameter overhead. The CTCI module yields moderate yet consistent metric gains through bidirectional fine-grained cross-task feature interaction, enhancing intrinsic feature correlation via spatial alignment and dynamic gating.
2. Pairwise combinations of the three core modules produce evident performance synergy beyond simple numerical superposition, attributed to their complementary functional attributes. The integration of TALA and MSTAF forms a complete workflow of feature embedding and decoding deviation calibration. The combination of TALA and CTCI alleviates feature aliasing and gradient conflict via task-aware modulation and cross-task interactive optimization. Merging MSTAF and CTCI strengthens multi-scale context fusion and inter-task semantic transmission. Nevertheless, dual-module combinations cannot cover all optimization dimensions, thus failing to reach the accuracy of the full integrated framework.
3. The shared Swin Transformer encoder provides a fundamental architecture for universal feature sharing and computational cost reduction, eliminating redundant feature extraction and lowering parameter occupancy and inference latency. Consistent performance gains on two datasets confirm that shared hierarchical features can satisfy heterogeneous optimization demands of interpolation and segmentation. Integrating the shared encoder with TALA, MSTAF and CTCI establishes a closed-loop learning pipeline covering task adaptive modulation, cross-level feature alignment and fine-grained cross-task interaction. This integrated mechanism enables mutual promotion between anatomical constraints from segmentation and spatiotemporal context from interpolation, achieving optimal performance among all ablation variants.

Joint multi-task learning realizes mutual performance promotion inherently: segmentation supplies anatomical prior to refine interpolation quality, while interpolation provides continuous spatiotemporal context to strengthen segmentation accuracy and inter-slice consistency. This mechanism works stably on both heart and lung datasets. Efficiency assessment indicates that the proposed lightweight framework delivers competitive inference speed and frame rate compared with cascaded schemes. Heavyweight dual-model combinations suffer from computational redundancy, while lightweight cascaded methods sacrifice task accuracy for speed. Our shared encoder design eliminates redundant feature extraction and achieves an optimal balance between prediction accuracy and computational efficiency, possessing good deployment compatibility for mid-to-low-end clinical computing devices and promising application value for routine batch medical image analysis.

### 5.2. Comparison with State of the Art

SOTA Models

1. ACVTT: Cross-view texture transfer for CT slice interpolation [4].
2. $I^{3}$Net: Inter-intra-slice network for medical slice synthesis [71].
3. Video Interp Net: Video frame interpolation for 3D tomography [72].
4. SFCLI-Net: Spatial-frequency collaborative CT slice interpolation [73].
5. SegMamba-V2: Mamba-based 3D medical image segmentation [74].
6. HiDiff: Hybrid diffusion medical image segmentation [75].
7. Anatomy-Aware Seg: CT airway tree segmentation with topology guidance [76].
8. SicTTA: Single-image test-time adaptation for segmentation [77].

We present quantitative comparisons between the proposed method and state-of-the-art models in Table 20, Table 21, Table 22 and Table 23.

1. Heart MRI Analysis: Existing state-of-the-art methods show competitive performance under single-task training settings on the cardiac MRI dataset. SFCLI-Net obtains marginally better SSIM value while showing unstable LPIPS performance with large standard deviation in interpolation experiments. SegMamba-V2 and HiDiff achieve competitive Dice and Precision scores in left atrium segmentation tasks. All referenced SOTA models are independently optimized for only one single task scenario. TASC-SwinMT attains the best PSNR and EdgeSSIM values among all interpolation methods. It achieves the highest IoU score in segmentation and maintains comparable Dice and Precision accuracy with advanced SOTA models while surpassing all conventional baseline networks.
2. Lung CT Analysis: The lung CT dataset involves irregular tumor contour features and raises higher difficulty for slice reconstruction and lesion segmentation tasks. Existing SOTA methods retain competitive advantages in their respective independent task domains. SFCLI-Net acquires the highest SSIM value in lung CT slice interpolation comparison. The proposed framework achieves optimal PSNR, EdgeSSIM and equivalent LPIPS performance against all contrast methods. It exceeds all SOTA models on IoU and Recall indicators for lung tumor segmentation, and maintains competitive Dice slightly lower than HiDiff yet higher than SegMamba-V2 while keeping Precision at a competitive level marginally below both models. The proposed framework presents stronger adaptive ability for complex irregular lesion structures than comparative models.
3. Comprehensive Multi-Task Advantage: Existing SOTA models only support independent single-task optimization and cannot finish interpolation and segmentation within one forward inference. Simply cascading separate SOTA interpolation and segmentation networks will introduce severe computational redundancy and resource waste. Such combined schemes inevitably increase inference delay, total parameter quantity and graphics memory occupation. The proposed framework employs a shared encoder to extract universal spatial features for dual tasks. It completes interpolation frame generation and segmentation mask prediction synchronously without repeated feature extraction procedures. The model maintains prediction accuracy competitive with mainstream SOTA methods and achieves superior multi-task performance while reducing computational overhead and accelerating inference efficiency. The balanced accuracy and efficiency enable the proposed framework to adapt better to clinical batch processing and real-time medical image analysis scenarios.

Computational Efficiency Comparison with SOTA Models

All efficiency experiments were run on a consistent hardware platform equipped with an NVIDIA RTX 4090 graphics card. We choose typical combined models from mainstream baseline networks and advanced interpolation and segmentation methods to conduct fair performance comparisons. The evaluation covers inference speed, frame processing rate, network parameters, graphics memory usage, computational complexity and model file size. Every quantitative result comes from the average value of five independent repeated tests. State-of-the-art single-task methods present excellent accuracy in independent tasks. The combination of these methods for dual-task processing creates heavy computational redundancy. All combined benchmark models spend more time on inference, process fewer frames per second and consume more computational resources than the proposed framework. The shared encoder structure of our method eliminates redundant feature extraction. This design achieves an excellent trade-off between multi-task performance and computational efficiency.

Table 24 and Table 25 present complete computational efficiency metrics of all tested models on the lung CT dataset.

### 5.3. Performance Superiority Analysis of the Proposed Multi-Task Learning Framework

The performance gap between TASC-SwinMT and the four comparative methods originates from fundamental differences in network architecture and cross-task learning mechanism. Conventional multi-task methods suffer from inherent structural defects when handling joint interpolation and segmentation of CT and MRI, while the proposed framework remedies these limitations via task-adaptive modulation, feature alignment and fine-grained cross-task fusion.

Direct feature concatenation merely superposes channel-wise features without calibrating distribution mismatch and semantic heterogeneity between two tasks. It cannot filter redundant features or relieve optimization gradient conflict, and fails to mine anatomical complementary relations, resulting in performance inferior to single-task baselines.

Shared-Bottom adopts hard parameter sharing for universal feature extraction and sets independent task heads. However, it lacks explicit cross-task interaction and feature alignment modules. The fixed shared structure cannot adapt to heterogeneous optimization demands of interpolation and segmentation, and cannot mitigate task gradient conflict, thus only maintaining performance comparable to single-task baselines.

Cross-task feature concatenation realizes multi-scale intermediate feature transmission and captures shallow cross-task correlations. Nevertheless, it remains limited to simple feature superposition, without frequency-domain modeling and bidirectional feature guidance. It lacks dynamic weight adjustment and distribution deviation correction, which restricts performance upper bound and only brings marginal improvement over baselines.

MoE enriches feature representation via multiple expert networks and integrates outputs through global gating. Its task-agnostic gating cannot allocate expert resources for specific tasks, and it has no targeted task modulation and cross-task fusion design. Each expert lacks optimization orientation for interpolation and segmentation, leading to limited performance gain.

Unlike the above methods, TASC-SwinMT establishes a complete multi-task collaborative learning paradigm. The shared Swin Transformer encoder realizes efficient universal feature sharing. The TALA module provides task-adaptive spatial and frequency-domain representation to compensate the task-agnostic shortcoming of traditional shared structures. The MSTAF module eliminates cross-level feature mismatch via bidirectional cross-attention and frequency enhancement. The CTCI module achieves fine-grained bidirectional cross-task interaction and dynamic gating fusion. Moreover, the learnable dynamic multi-task loss adaptively balances heterogeneous task optimization and alleviates gradient conflict and training bias. The organic combination of these designs fully excavates the mutual promotion between interpolation and segmentation, and achieves prominent performance superiority over all mainstream multi-task methods.

### 5.4. Analysis of Multi-Task Loss Strategy Performance and Training Dynamics Differences

This subsection conducts a in-depth analysis of the performance differences between the three multi-task balancing strategies, and explores the underlying reasons for the distinct training dynamics observed in the experiments.

The core design differences between the three strategies are the fundamental cause of the final performance divergence. Grad-Norm adopts a gradient amplitude normalization mechanism, which only focuses on unifying the gradient scaling of different tasks to maintain stable gradient propagation, without establishing any constraints between task-wise feature contribution and actual task optimization status. Such a mechanism lacks task-level adaptive guidance, so the model naturally leans toward the interpolation task with simpler optimization landscape and more stable gradient feedback, resulting in long-term bias toward interpolation and insufficient segmentation optimization. DWA relies on dynamic gradient weight scheduling based on batch-level global statistics, which also ignores task-specific feature representation learning and real-time optimization feedback. Without explicit constraints on dual-task feature allocation, DWA similarly prioritizes the interpolation task and fails to achieve homogeneous optimization balance between heterogeneous tasks. In contrast, the proposed dynamic loss strategy integrates learnable adaptive weights with structured regularization constraints, and performs synchronous weight adjustment by coupling task feature contribution and real-time optimization feedback. It works with the cross-task feature interaction modules to promote complementary representation learning, thus breaking the task bias issue and achieving coordinated optimization between dual tasks without sacrificing either task performance.

The differences in training dynamics are directly determined by the intrinsic optimization logic of the three strategies. DWA allocates dominant optimization resources to the interpolation task at the early training stage, which leads to rapid fitting of the interpolation objective but completely suppresses the initialization of segmentation learning. Since subsequent gradient-based adjustment cannot reverse the early learning bias, the model suffers from sustained optimization oscillation and slow segmentation convergence throughout training. Grad-Norm maintains a relatively gentle early convergence trend for the interpolation task, but its pure gradient-driven adjustment mechanism cannot break the inherent optimization bottleneck of the segmentation task. The model remains trapped in the local optimum dominated by the interpolation task, resulting in limited upper bound of segmentation performance. The proposed method adopts a balanced initialization strategy for dual-task weights in the early stage, forcing the model to first capture universal shared anatomical features. As training proceeds, it dynamically adjusts the weight distribution to focus on fine-grained task-specific optimization, which yields a more stable and balanced convergence trajectory with smaller fluctuations and better global optimization.

The feature contribution patterns further reveal the essential mechanism behind performance gaps. The stable segmentation feature contribution in Grad-Norm and DWA is not a sign of robust learning, but a result of extreme long-term imbalance in dual-task feature allocation. The segmentation task is persistently deprived of sufficient feature learning resources, making it unable to acquire discriminative target-aware representations. Such overly stable yet severely skewed feature distribution fundamentally limits the upper bound of segmentation performance. The proposed method introduces moderate and adaptive oscillation into segmentation feature contribution, which dynamically adjusts the feature allocation ratio according to the real-time optimization state of the segmentation task. This adaptive oscillatory mechanism continuously strengthens the feature extraction capability dedicated to segmentation targets, effectively breaks through the performance ceiling imposed by the rigid stable allocation paradigm, and unlocks higher optimization potential for the segmentation task.

From the perspective of clinical application, the proposed method has more significant practical value. Clinical diagnosis scenarios have higher requirements for the accuracy of segmentation tasks, which directly affect the accuracy of lesion localization, quantitative evaluation and treatment planning. The single BCE loss fits well with our multi-task structure and effectively relieves class imbalance issues via cross-task constraint and multi-scale feature enhancement. This simple loss setting also simplifies optimization and ensures stable model convergence. The proposed method maintains stable interpolation reconstruction quality while significantly improving the segmentation performance, which is more in line with the actual needs of clinical applications. At the same time, the stable training process and good convergence effect also make the proposed method have better robustness and generalization ability in the deployment of actual clinical data, avoiding the model performance instability caused by training process fluctuations.

### 5.5. Generalization Evaluation on External Datasets

To further validate the out-of-distribution generalization ability of the proposed TASC-SwinMT framework, additional experiments are conducted on two external public 3D CT datasets independent of the original MSD heart and lung datasets. The first 3D liver tumor CT dataset with 123 patient samples is obtained from Kaggle at https://www.kaggle.com/datasets/prathamgrover/3d-liver-segmentation (accessed on 10 May 2026), and it provides annotated volumetric scans for liver tumor segmentation and inter-slice interpolation evaluation. The second COVID-19 chest CT dataset including 20 patient scans is collected from Kaggle at https://www.kaggle.com/datasets/andrewmvd/covid19-ct-scans (accessed on 11 May 2026), which contains expert-labelled lung and infection segmentation masks and represents a typical clinical scenario of thoracic lesion analysis with ambiguous tissue boundaries. Both datasets are partitioned into training set, validation set and test set following a strict patient-level division ratio of 7:1:2 to avoid slice-level data leakage and ensure experimental authenticity. All data preprocessing strategies and model training configurations remain completely consistent with those adopted on the original MSD datasets.

**Figure 16:**
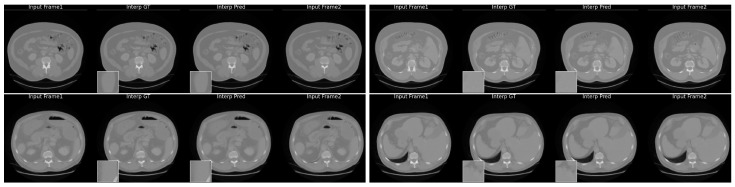
Qualitative visualization of liver CT inter-slice interpolation.

**Figure 17:**
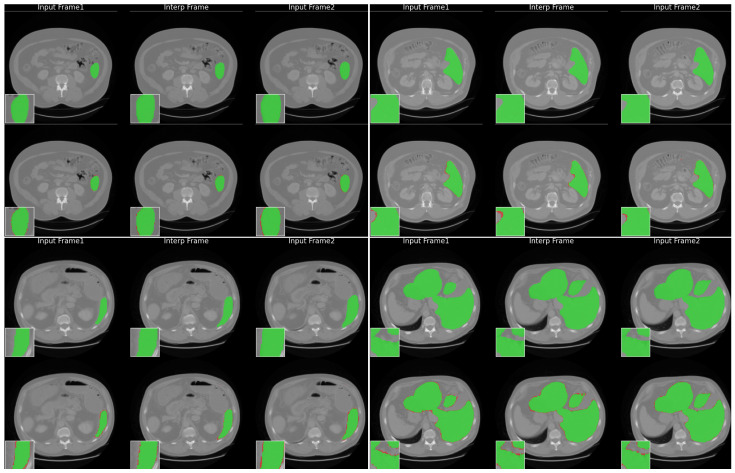
Qualitative visualization of liver tumor segmentation.

**Figure 18:**
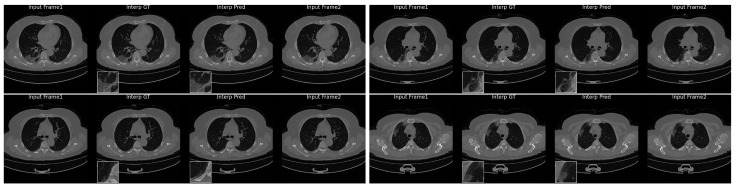
Qualitative visualization of COVID-19 CT inter-slice interpolation.

**Figure 19:**
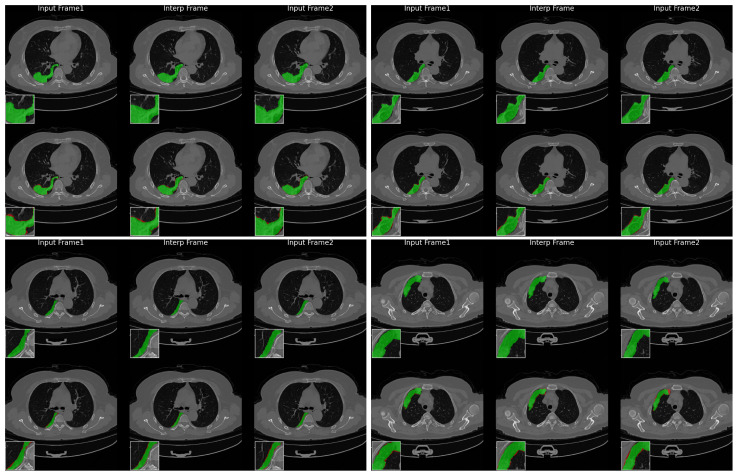
Qualitative visualization of COVID-19 lung infection segmentation.

We select four representative slices from each dataset to visualize the interpolation and segmentation results. Four representative sample slices are selected from each external dataset for qualitative visualization. All interpolation results are arranged in one row with four subgraphs. The first and fourth subgraphs correspond to input frames, the second subgraph denotes the real intermediate frame, and the third subgraph is the intermediate frame predicted by the model. All segmentation results adopt a layout of two rows and three columns. The first row displays two input frames together with their corresponding ground truth segmentation labels, as well as the real intermediate frame and its annotation. The second row presents predicted segmentation masks of two input frames, and the segmentation output obtained from the predicted intermediate frame. In segmentation visualization, green areas represent correct segmentation regions, and red areas mark misdiagnosis and missed diagnosis regions. Figure 16 and Figure 17 show qualitative results on the Liver dataset, Figure 18 and Figure 19 on the COVID19 dataset.

Table 26 summarizes the quantitative interpolation performance on liver CT and COVID-19 CT datasets. The proposed method maintains high structural reconstruction and perceptual similarity metrics on both external datasets.

Table 27 reports the segmentation quantitative results of the two external datasets.

Table 28 and Table 29 list the computational efficiency metrics of the proposed framework on two external datasets, with each table containing 3 core indicators for comprehensive evaluation. Table 28 presents the static model complexity indicators, including parameter scale, GPU memory occupation and computational complexity, which remain almost consistent across the two datasets with negligible numerical fluctuation, as the network structure and parameter scale are fixed during inference. Table 29 reports the dynamic inference performance indicators, including average inference time, average frame rate and model storage size. The two datasets exhibit highly close inference efficiency, with negligible gaps in inference time and frame rate, which is attributed to the unified network structure and optimized feature extraction strategy adopted by the framework. The stable performance on both datasets confirms the strong adaptability of the model to different clinical CT imaging scenarios.

Combined with the above quantitative and qualitative results covering reconstruction, segmentation and temporal consistency indicators, the proposed TASC-SwinMT framework exhibits stable cross-dataset generalization capability, while limitations remain to be addressed. For the liver CT dataset, the interpolation visualization shows that the interpolated frame maintains overall anatomical consistency with the input frames, with smooth tissue transitions and no obvious structural distortion and steady temporal consistency across consecutive slices. Quantitatively, the SSIM reaches 0.9655 ± 0.0025 and PSNR reaches 38.7054 ± 0.25 dB, indicating good pixel-level and structural reconstruction quality. However, the EdgeSSIM is only 0.8589 ± 0.0032, which is significantly lower than the overall SSIM. This gap is visually confirmed in the segmentation results: while the Dice coefficient reaches 0.9653 ± 0.0042, the red-marked edge regions in the zoomed views reveal segmentation errors at the boundary between the liver and surrounding abdominal structures. The model struggles to capture fine, low-contrast contours, leading to incomplete delineation of the liver edge in some cases. This limitation is likely due to the complex background interference in abdominal CT scans, where the liver borders are often blurred by adjacent organs and varying tissue densities.

For the COVID-19 CT dataset, the segmentation task presents greater inherent challenges: infected regions are often irregularly shaped, with low contrast against normal lung parenchyma and indistinct borders, making them harder to segment than the relatively homogeneous liver tissue. Quantitatively, the Dice coefficient of 0.9046 ± 0.0053 reflects reasonable overall segmentation performance, but the gap between Precision (0.9169 ± 0.0049) and Recall (0.8970 ± 0.0058) reveals a clear performance imbalance. The higher Precision indicates the model effectively reduces false positive predictions and avoids misclassifying normal lung tissue as infected regions, while the lower Recall reflects the model cannot completely capture all real infection areas, especially in regions with ambiguous and blurred lesion boundaries. This limitation is directly visible in the segmentation visualization results: while the main body of the infection regions is accurately segmented, the red-marked error regions are concentrated at the lesion periphery, particularly where infected tissue blends gradually with normal lung parenchyma. The model struggles to resolve fine, low-contrast edges, leading to partial under-segmentation of infection margins and occasional jagged contours that do not align with the true lesion shape. These errors are more pronounced in cases where the infection abuts the mediastinum or pleura, where tissue contrast is further reduced by adjacent anatomical structures.

In terms of computational efficiency, the model maintains a fixed parameter scale of 33.36 million and GPU memory occupation of 2252.11 MB on both datasets, which is far lower than the cascaded single-task SOTA models in the previous experiments. The inference speed of liver CT is 56.65 ms per sample with 35.31 FPS, and COVID-19 CT is 56.20 ms per sample with 35.60 FPS, both maintaining a real-time processing level for 3D liver CT and 2D COVID-19 CT slices. The shared Swin Transformer encoder extracts universal anatomical spatial features applicable to different abdominal and thoracic CT scenarios. The designed TALA, MSTAF and CTCI modules enhance the adaptive feature modulation and cross-task interaction ability, enabling the model to adapt to diversified imaging characteristics and lesion morphological distributions. Meanwhile, the stable computational efficiency across different datasets further verifies that the proposed multi-task framework possesses good practical deployment potential for multi-scene medical CT interpolation and segmentation tasks.

### 5.6. Interpretability Visualization Result Analysis

Feature visualization experiments on heart and lung datasets confirm the proposed framework possesses stronger feature representation ability and clear mechanism interpretability. Cross-task collaborative attention maps of TASC-SwinMT can accurately activate task-related anatomical regions. The model suppresses response interference from irrelevant background and peripheral normal tissues. Baseline methods generate discrete and disconnected attention activation areas and cannot precisely locate target lesion and tissue regions.

Anatomical edge reconstruction features extracted by the proposed model can restore complete and smooth tissue boundary contours. Stable reconstruction performance can be maintained in low-contrast imaging areas and tissue overlapping positions. Baseline models show obvious edge blurring and cannot distinguish target tissue boundaries from adjacent surrounding structures. Frequency-domain global feature maps of the proposed method can capture complete multi-level anatomical structural information. Baseline methods show disordered feature distribution and lack effective modeling ability for global anatomical structure. Segmentation boundary attention distribution of the proposed model keeps high consistency with manual annotated ground truth contours. Baseline models generate obvious position offset and contour deviation in boundary localization.

Stable performance advantages on two experimental datasets validate the rationality of the three core module design strategies. The CTCI module guides network attention to focus on clinically significant regions through bidirectional cross-task feature interaction. The TALA module integrates frequency-domain global modeling to balance local detail feature extraction and long-range global structural perception. The MSTAF module eliminates feature distribution deviation between encoding and decoding stages and ensures structural feature continuity across multi-scale layers.

### 5.7. Limitations

1. Despite undergoing extreme lightweight optimization, the proposed framework has a compact parameter size of only 33.3589 million parameters and delivers favorable inference efficiency on the NVIDIA RTX 4090 platform: the average inference latency is 58.82 ms for lung CT samples, with comparable latency for cardiac MRI samples and slightly lower latency on external generalized CT datasets. Nevertheless, further computational efficiency optimization can still be implemented to better satisfy the strict requirements of intraoperative real-time diagnosis guidance and batch processing of large-scale clinical datasets. The main computational complexity originates from the deeply stacked Swin Transformer encoder and dual task-specific decoders deployed for fine-grained anatomical feature extraction, and we will continue to streamline this structure in follow-up research to pursue higher efficiency while maintaining model performance.
2. Although this study has conducted additional generalization experiments on external datasets including 3D liver tumor CT and COVID-19 chest CT datasets, the model is still mainly trained and evaluated on limited public datasets from the Medical Segmentation Decathlon (MSD) with unified imaging protocols and standardized annotation criteria. It has not been tested on more clinically specific and high-difficulty datasets involving complex lesions, rare diseases, severe imaging artifacts, and heterogeneous multi-center clinical data collected from different scanning equipment, diverse patient cohorts and independent manual annotation systems. As a result, the model still cannot satisfy all the complex and diverse requirements of practical clinical applications, and further research is needed to continuously improve its generalization ability to adapt to more complicated clinical scenarios.
3. This framework does not integrate explicit physiological motion modeling mechanisms to characterize cardiac contraction patterns and lung respiratory movements. The interpolation results may produce anatomically unreasonable tissue structures for image frames captured at typical physiological motion phases.
4. The designed dynamic multi-task loss only optimizes task weight allocation at the global batch level throughout the training process. The loss strategy fails to achieve independent adaptive weight adjustment for each individual sample. It is difficult to realize refined optimization balance for samples with variable anatomical morphologies and blurred lesion boundary characteristics.
5. The feasibility of the proposed model architecture has not been verified on multi-center datasets equipped with hierarchical classification diagnosis labels. Current experimental datasets only contain segmentation annotation information and lack graded clinical diagnosis labels corresponding to lesion severity and disease staging. The absence of validation on label-rich multi-center data limits the comprehensive clinical application value of the framework. We will supplement this verification work in future research to further improve the clinical practicability and robustness of the model.

### 5.8. Future Work

1. Model lightweight: Subsequent research will adopt knowledge distillation channel pruning and INT8 model quantization strategies to compress total parameters below 15 million. Inference speed will be optimized to no more than 0.05 s per sample to support real-time clinical deployment while retaining over 95% of the original model performance.
2. Multi-dataset validation: Follow-up work will collect multi-center clinical thoracic imaging data covering diversified scanning parameters patient groups and lesion distribution types. Domain adaptation methods including adversarial training and image style transfer will be adopted to alleviate data distribution difference and verify model generalization in practical clinical scenarios.
3. Motion model fusion: Subsequent studies will fuse deformable registration based physiological motion models to learn inherent movement rules of lung respiration and cardiac contraction. The embedded motion prior will improve anatomical rationality and physiological consistency of generated intermediate interpolation frames.
4. Adaptive loss weights: Future research will design reinforcement learning based dynamic loss adjustment strategies taking Deep Q-Network as the basic framework. Independent loss weights will be optimized for each single sample according to lesion scale imaging quality and anatomical complexity to further promote model adaptability on heterogeneous medical data.
5. Clinical system development: An end-to-end intelligent medical analysis platform will be constructed to integrate image import preprocessing joint interpolation segmentation and lesion quantitative calculation. Automatic measurement of left atrium volume and lung tumor size will be realized with intuitive graphical interaction interface to assist clinical diagnosis and individualized treatment planning.

## 6. Conclusions

This paper introduces a novel SwinUNet-based multi-task learning framework for the joint interpolation and semantic segmentation of CT and MRI images. The framework is applied to left atrium segmentation in cardiac MRI and lung tumor segmentation in thoracic CT. To address key challenges in joint modeling, including spatiotemporal feature fusion and balance between heterogeneous tasks, the framework incorporates three core components: a shared encoder and dual decoders that enable efficient feature sharing and mutual task enhancement, three innovative modules including TALA, CTCI and MSTAF that optimize feature adaptation and cross-task collaboration, and a dynamic multi-task loss with learnable weights that automatically balances optimization between pixel-level reconstruction and classification objectives.

Extensive validations on the public MSD Task02_Heart and Task06_Lung datasets confirm the competitive performance on PSNR, SSIM, EdgeSSIM, LPIPS, Dice, IoU, Precision and Recall as well as strong generalization of the proposed TASC-SwinMT framework.

The proposed multi-task framework breaks the bottleneck of traditional single-task independent processing, and realizes efficient feature sharing and performance mutual promotion between lung CT and cardiac MRI interpolation and segmentation. Segmentation provides anatomical constraints to optimize interpolation quality, and interpolation supplies spatiotemporal context to improve segmentation accuracy. This framework offers a reliable technical scheme for efficient and precise disease diagnosis based on CT and MRI. Future research will focus on model lightweighting, multi-center clinical dataset validation and organ motion model embedding to further improve clinical practicality.

## Figures and Tables

**Table 1:** Key feature comparison of representative related methods.

Method Category	Core Feature	Main Limitation
Traditional Interpolation	Linear/B-spline/Fourier reconstruction	Poor anatomical structure fitting, blurry detail
Deep Interpolation	Motion/texture based single-task interpolation	No segmentation constraint, lack frequency modeling
CNN Segmentation	U-Net variant local semantic extraction	Insufficient long-range dependency modeling
Transformer Segmentation	Global sequence feature modeling	Task-agnostic design for multi-task scenario
General MTL	Shared feature learning for correlated tasks	Simple fusion, fixed loss weight, no interpolation-segmentation joint design
Swin Medical Vision	Hierarchical window attention feature extraction	Lack task-adaptive module and frequency-domain embedding
Proposed TASC-SwinMT	Shared encoder + task-adaptive module + cross-task interaction + dynamic loss	Oriented for CT and MRI joint interpolation-segmentation

**Table 2:** Feature dimension evolution across encoder stages.

Stage	Input Dimension	Output Dimension	Number of Blocks	Number of Heads
1	B×96×128×128	B×96×128×128	1	2
2	B×96×128×128	B×192×64×64	1	4
3	B×192×64×64	B×384×32×32	1	8
4	B×384×32×32	B×768×16×16	1	16

**Table 3:** Key symbol definition.

Symbol	Description	Unit/Dimension
$X_{i}$	The *i*-th input frames CT for lung and MRI for heart	$R^{B\times 1\times H\times W}$
${\hat{X}}_{2}$	Predicted intermediate frame from image interpolation	$R^{B\times 1\times H\times W}$
$Y_{i}$	Ground truth mask for medical image segmentation	$R^{B\times 1\times H\times W}$
${\hat{Y}}_{i}$	Predicted mask from medical image segmentation	$R^{B\times 1\times H\times W}$
TALA	Task-Aware Lightweight Adapter module	-
MSTAF	Multi-Scale Task Alignment Fusion module	-
CTCI	Cross-Task Collaborative Interaction module	-
$\alpha_{\mathrm{tala}}$	Channel attention weight in the TALA module	$R^{B\times C\times 1\times 1}$
α	Balance parameter in hybrid interpolation loss	-
$W_{1},W_{2}$	Dynamic task fusion weights in the MSTAF module	Scalar
$\theta_{1},\theta_{2}$	Learnable parameters for dynamic multi-task loss balancing	$R^{2}$
$W_{\mathrm{soft}}$	Learnable weight projection matrix in MSTAF dynamic gating	-
*B*	Batch size in model training	2
*C*	Channel dimension of intermediate feature maps	-
H,W	Height and width of input CT images	512,512
$N_{h}$	Number of multi-head attention heads	-
$d_{k}$	Dimension of each attention head	-

**Table 4:** Heart dataset: interpolation baseline quantitative results.

Model	PSNR (dB)	SSIM	LPIPS	EdgeSSIM
SwinUNet-Interp	36.28 ± 0.47 ***	0.952 ± 0.0021 ***	0.114 ± 0.003 ***	0.938 ± 0.002 ***
TransUNet-Interp	37.51 ± 0.36 ***	0.947 ± 0.0011 ***	0.122 ± 0.002 ***	0.929 ± 0.003 ***
VTN-Interp	35.84 ± 0.42 ***	0.958 ± 0.0017 ***	0.107 ± 0.003 ***	0.945 ± 0.001 ***
Ours	**41.50 ± 0.20**	**0.990 ± 0.0003**	**0.051 ± 0.001**	**0.988 ± 0.002**

**Table 5:** Heart dataset: segmentation baseline quantitative results.

Model	Dice	IoU	Precision	Recall
nnU-Net-Seg	0.904 ± 0.015 ***	0.871 ± 0.013 ***	0.899 ± 0.017 ***	0.907 ± 0.014 ***
UNet++-Seg	0.887 ± 0.012 ***	0.852 ± 0.016 ***	0.882 ± 0.014 ***	0.891 ± 0.015 ***
Swin UNETR-Seg	0.915 ± 0.016 **	0.883 ± 0.012 ***	0.911 ± 0.013 ***	0.918 ± 0.016 **
Ours	**0.967 ± 0.005**	**0.940 ± 0.007**	**0.969 ± 0.007**	**0.968 ± 0.005**

**Table 6:** Lung dataset: interpolation baseline quantitative results.

Model	PSNR (dB)	SSIM	LPIPS	EdgeSSIM
SwinUNet-Interp	35.71 ± 0.47 ***	0.940 ± 0.0021 ***	0.119 ± 0.003 ***	0.926 ± 0.002 ***
TransUNet-Interp	36.94 ± 0.36 ***	0.935 ± 0.0011 ***	0.127 ± 0.002 ***	0.917 ± 0.003 ***
VTN-Interp	35.27 ± 0.42 ***	0.946 ± 0.0017 ***	0.112 ± 0.003 ***	0.933 ± 0.001 ***
Ours	**40.76 ± 0.38**	**0.974 ± 0.0019**	**0.058 ± 0.002**	**0.969 ± 0.002**

**Table 7:** Lung dataset: segmentation baseline quantitative results.

Model	Dice	IoU	Precision	Recall
nnU-Net-Seg	0.874 ± 0.015 ***	0.821 ± 0.013 ***	0.870 ± 0.017 ***	0.877 ± 0.014 ***
UNet++-Seg	0.857 ± 0.012 ***	0.802 ± 0.016 ***	0.853 ± 0.014 ***	0.861 ± 0.015 ***
Swin UNETR-Seg	0.885 ± 0.016 **	0.833 ± 0.012 **	0.881 ± 0.013 **	0.888 ± 0.016 **
Ours	**0.926 ± 0.014**	**0.869 ± 0.013**	**0.925 ± 0.015**	**0.928 ± 0.014**

**Table 8:** Heart dataset: ablation results of model variants.

Model	PSNR (dB)	SSIM	Dice	Recall
IndEnc	38.33 ± 0.52 ***	0.961 ± 0.0041 ***	0.912 ± 0.024 **	0.915 ± 0.019 **
+TALA	39.46 ± 0.39 ***	0.970 ± 0.0027 ***	0.935 ± 0.016 **	0.938 ± 0.013 **
+CTCI	39.12 ± 0.67 ***	0.968 ± 0.0033 ***	0.930 ± 0.021	0.932 ± 0.017 **
+MSTAF	39.68 ± 0.34 ***	0.972 ± 0.0022 ***	0.941 ± 0.012 **	0.943 ± 0.010 **
+TALA+CTCI	40.25 ± 0.45 **	0.979 ± 0.0018 ***	0.952 ± 0.009 *	0.954 ± 0.008 *
+TALA+MSTAF	40.61 ± 0.30 ***	0.983 ± 0.0013 ***	0.958 ± 0.007	0.959 ± 0.006 *
+CTCI+MSTAF	40.42 ± 0.58 *	0.981 ± 0.0015 ***	0.955 ± 0.011	0.957 ± 0.009
Ours	**41.50 ± 0.20**	**0.990 ± 0.0003**	**0.967 ± 0.005**	**0.968 ± 0.005**

**Table 9:** Lung dataset: ablation results of model variants.

Model	PSNR (dB)	SSIM	Dice	Recall
IndEnc	37.76 ± 0.56 ***	0.949 ± 0.0043 ***	0.875 ± 0.030 *	0.879 ± 0.026 **
+TALA	38.85 ± 0.42 ***	0.958 ± 0.0031 ***	0.892 ± 0.022 *	0.895 ± 0.018 *
+CTCI	38.51 ± 0.61 ***	0.955 ± 0.0036 ***	0.888 ± 0.027 *	0.891 ± 0.023 *
+MSTAF	39.07 ± 0.37 ***	0.960 ± 0.0026 ***	0.899 ± 0.019 *	0.902 ± 0.015 *
+TALA+CTCI	39.64 ± 0.49 **	0.965 ± 0.0021 ***	0.910 ± 0.017	0.913 ± 0.014
+TALA+MSTAF	40.12 ± 0.33 *	0.969 ± 0.0017 **	0.918 ± 0.020	0.920 ± 0.016
+CTCI+MSTAF	39.89 ± 0.53 *	0.967 ± 0.0024 **	0.914 ± 0.018	0.916 ± 0.013
Ours	**40.76 ± 0.38**	**0.974 ± 0.0019**	**0.926 ± 0.014**	**0.928 ± 0.014**

**Table 10:** Loss weight ablation (Heart dataset).

Weight	PSNR (dB)	Dice	Total Loss
0.3/0.7	40.61 ± 0.42 **	0.955 ± 0.009 *	0.00368 ± 0.0008 **
0.4/0.6	41.12 ± 0.25 *	0.961 ± 0.006	0.00287 ± 0.00010 ***
0.5/0.5	41.03 ± 0.31 *	0.954 ± 0.007 *	0.00335 ± 0.00011 ***
Ours	**41.50 ± 0.20**	**0.967 ± 0.005**	**0.00204 ± 0.00008**

**Table 11:** Expanded ablation studies: component contribution (Heart dataset).

Component	ΔPSNR (dB)	ΔSSIM (%)	ΔDice (%)	ΔRecall (%)
TALA	1.13	0.90	2.30	2.30
CTCI	0.79	0.70	1.80	1.70
MSTAF	1.35	1.10	2.90	2.80
TALA+CTCI	1.92	1.80	4.00	3.90
TALA+MSTAF	2.28	2.20	4.60	4.40
CTCI+MSTAF	2.09	2.00	4.30	4.20
Full Framework	**3.17**	**2.90**	**5.50**	**5.30**

**Table 12:** Expanded ablation studies: component contribution (Lung dataset).

Component	ΔPSNR (dB)	ΔSSIM (%)	ΔDice (%)	ΔRecall (%)
TALA	1.09	0.90	1.70	1.60
CTCI	0.75	0.60	1.30	1.20
MSTAF	1.31	1.10	2.40	2.30
TALA+CTCI	1.88	1.60	3.50	3.40
TALA+MSTAF	2.36	2.00	4.30	4.10
CTCI+MSTAF	2.13	1.80	3.90	3.80
Full Framework	**3.00**	**2.50**	**5.10**	**4.90**

**Table 13:** Interpolation performance comparison of different multi-task learning methods.

Method	PSNR (dB)	SSIM	LPIPS	EdgeSSIM
Direct Concatenation	35.42 ± 0.35 ***	0.936 ± 0.0026 ***	0.125 ± 0.002 ***	0.923 ± 0.003 ***
Shared-Bottom	36.35 ± 0.32 ***	0.945 ± 0.0015 ***	0.116 ± 0.003 ***	0.934 ± 0.004 ***
Cross-Task Concatenation	37.68 ± 0.53 ***	0.954 ± 0.0018 ***	0.103 ± 0.002 ***	0.946 ± 0.003 ***
MoE	37.92 ± 0.41 ***	0.957 ± 0.0012 ***	0.098 ± 0.004 ***	0.949 ± 0.001 ***
Ours	**41.50 ± 0.20**	**0.990 ± 0.0003**	**0.051 ± 0.001**	**0.988 ± 0.002**

**Table 14:** Segmentation performance comparison of different multi-task learning methods.

Method	Dice	IoU	Precision	Recall
Direct Concatenation	0.871 ± 0.009 ***	0.818 ± 0.017 ***	0.868 ± 0.019 ***	0.873 ± 0.014 ***
Shared-Bottom	0.883 ± 0.014 ***	0.830 ± 0.012 ***	0.880 ± 0.015 ***	0.885 ± 0.016 ***
Cross-Task Concatenation	0.896 ± 0.011 ***	0.842 ± 0.015 ***	0.893 ± 0.012 ***	0.898 ± 0.023 ***
MoE	0.901 ± 0.018 ***	0.847 ± 0.010 ***	0.898 ± 0.010 ***	0.903 ± 0.009 ***
Ours	**0.967 ± 0.005**	**0.940 ± 0.007**	**0.969 ± 0.007**	**0.968 ± 0.005**

**Table 15:** Quantitative comparison of multi-task balancing strategies on Cardiac dataset.

Strategy	PSNR (dB)	SSIM	Dice	Recall
Grad-Norm	42.06 ± 0.22	0.9909 ± 0.0003	0.920 ± 0.007 ***	0.918 ± 0.005 ***
DWA	**42.76 ± 0.14**	**0.9910 ± 0.0002**	0.932 ± 0.004 ***	0.929 ± 0.004 ***
Ours (Dynamic Loss)	41.50 ± 0.20	0.9904 ± 0.0002	**0.967 ± 0.005**	**0.968 ± 0.005**

**Table 16:** Heart dataset: interpolation metrics (single-task vs. multi-task).

Model	PSNR (dB)	SSIM	LPIPS	EdgeSSIM
TASC-Interp	39.25 ± 0.28 ***	0.971 ± 0.0008 ***	0.078 ± 0.002 ***	0.965 ± 0.0005 ***
Ours-MT	**41.50 ± 0.20**	**0.990 ± 0.0003**	**0.051 ± 0.001**	**0.988 ± 0.002**

**Table 17:** Heart dataset: segmentation metrics (single-task vs. multi-task).

Model	Dice	IoU	Precision	Recall	TCS
TASC-Seg	0.942 ± 0.008 ***	0.915 ± 0.009 **	0.945 ± 0.006 ***	0.943 ± 0.007 ***	0.921 ± 0.004 ***
Ours-MT	**0.967 ± 0.005**	**0.940 ± 0.007**	**0.969 ± 0.007**	**0.968 ± 0.005**	**0.976 ± 0.002**

**Table 18:** Lung dataset: interpolation metrics (single-task vs. multi-task).

Model	PSNR (dB)	SSIM	LPIPS	EdgeSSIM
TASC-Interp	38.42 ± 0.35 ***	0.953 ± 0.0015 ***	0.089 ± 0.002 ***	0.947 ± 0.0018 ***
Ours-MT	**40.76 ± 0.38**	**0.974 ± 0.0019**	**0.058 ± 0.002**	**0.969 ± 0.002**

**Table 19:** Lung dataset: segmentation metrics (single-task vs. multi-task).

Model	Dice	IoU	Precision	Recall	TCS
TASC-Seg	0.903 ± 0.011 *	0.846 ± 0.012 *	0.901 ± 0.013 *	0.905 ± 0.012 *	0.908 ± 0.005 ***
Ours-MT	**0.926 ± 0.014**	**0.869 ± 0.013**	**0.925 ± 0.015**	**0.928 ± 0.014**	**0.963 ± 0.003**

**Table 20:** Heart dataset: interpolation SOTA quantitative results.

Model	PSNR (dB)	SSIM	LPIPS	EdgeSSIM
ACVTT	39.42 ± 0.32 ***	0.976 ± 0.0016 ***	0.068 ± 0.008 **	0.972 ± 0.008 **
$I^{3}$Net	39.85 ± 0.28 ***	0.981 ± 0.0012 ***	0.062 ± 0.007 *	0.978 ± 0.006 *
Video Interp Net	40.21 ± 0.34 ***	0.985 ± 0.0010 ***	0.058 ± 0.011	0.982 ± 0.005
SFCLI-Net	41.43 ± 0.19	**0.991 ± 0.0007**	**0.048 ± 0.009**	0.984 ± 0.010
Ours	**41.50 ± 0.20**	0.990 ± 0.0003	0.051 ± 0.001	**0.988 ± 0.002**

**Table 21:** Heart dataset: segmentation SOTA quantitative results.

Model	Dice	IoU	Precision	Recall
SegMamba-V2	0.965 ± 0.014	0.936 ± 0.015	**0.973 ± 0.013**	0.961 ± 0.013
HiDiff	**0.969 ± 0.011**	0.938 ± 0.019	0.965 ± 0.012	**0.969 ± 0.017**
Anatomy-Aware Seg	0.958 ± 0.022	0.931 ± 0.014	0.960 ± 0.020	0.959 ± 0.016
SicTTA	0.955 ± 0.008 *	0.928 ± 0.010	0.957 ± 0.013	0.956 ± 0.012
Ours	0.967 ± 0.005	**0.940 ± 0.007**	0.969 ± 0.007	0.968 ± 0.005

**Table 22:** Lung dataset: interpolation SOTA quantitative results.

Model	PSNR (dB)	SSIM	LPIPS	EdgeSSIM
ACVTT	38.65 ± 0.36 ***	0.959 ± 0.0021 ***	0.074 ± 0.007 **	0.953 ± 0.008 **
$I^{3}$Net	39.12 ± 0.31 ***	0.964 ± 0.0014 ***	0.069 ± 0.005 **	0.958 ± 0.006 *
Video Interp Net	39.58 ± 0.37 **	0.968 ± 0.0018 ***	0.065 ± 0.004 *	0.963 ± 0.012
SFCLI-Net	40.71 ± 0.26	**0.975 ± 0.0013**	**0.058 ± 0.005**	0.968 ± 0.005
Ours	**40.76 ± 0.38**	0.974 ± 0.0019	**0.058 ± 0.002**	**0.969 ± 0.002**

**Table 23:** Lung dataset: segmentation SOTA quantitative results.

Model	Dice	IoU	Precision	Recall
SegMamba-V2	0.922 ± 0.016	0.861 ± 0.019	**0.933 ± 0.021**	0.924 ± 0.023
HiDiff	**0.929 ± 0.021**	0.865 ± 0.021	0.930 ± 0.023	0.926 ± 0.024
Anatomy-Aware Seg	0.921 ± 0.023	0.864 ± 0.026	0.920 ± 0.028	0.922 ± 0.018
SicTTA	0.918 ± 0.029	0.861 ± 0.031	0.917 ± 0.026	0.919 ± 0.032
Ours	0.926 ± 0.014	**0.869 ± 0.013**	0.925 ± 0.015	**0.928 ± 0.014**

**Table 24:** Computational efficiency of speed and parameter quantification on Lung CT dataset.

Model	Avg_Infer_Time (ms)	Avg_FPS	Parameters (M)
SwinUNet + nnU-Net	72.4835 ± 0.3625	24.8635 ± 0.2947	186.7251 ± 0.0000
TransUNet + UNETR	68.7526 ± 0.2531	27.9526 ± 0.2615	142.3685 ± 0.0000
VTN + UNet++	63.9418 ± 0.1428	30.2745 ± 0.3526	68.5122 ± 0.0000
ACVTT + SegMamba-V2	86.3745 ± 0.4836	19.6428 ± 0.2532	235.4126 ± 0.0000
$I^{3}$Net + HiDiff	81.2634 ± 0.4428	21.5836 ± 0.2746	218.6573 ± 0.0000
VideoNet+Anatomy-Aware	75.8427 ± 0.4135	23.4752 ± 0.2689	195.3248 ± 0.0000
SFCLI-Net + SicTTA	78.5316 ± 0.4267	22.3641 ± 0.2634	207.8965 ± 0.0000
Ours	**58.8218 ± 0.2153**	**34.1846 ± 0.1862**	**33.3589 ± 0.0000**

**Table 25:** Computational efficiency of memory and complexity evaluation on Lung CT dataset.

Model	Memory (MB)	FLOPs (G)	Model Size (MB)
SwinUNet + nnU-Net	2896.5327 ± 1.4236	489.2243 ± 0.0000	512.4637 ± 0.0000
TransUNet + UNETR	2643.7418 ± 1.2531	396.7118 ± 0.0000	468.5726 ± 0.0000
VTN + UNet++	2487.6235 ± 0.8625	187.3359 ± 0.0000	426.7418 ± 0.0000
ACVTT + SegMamba-V2	3952.8416 ± 1.8624	658.3215 ± 0.0000	648.7532 ± 0.0000
$I^{3}$Net + HiDiff	3726.7325 ± 1.7235	612.5894 ± 0.0000	601.4627 ± 0.0000
VideoNet+Anatomy-Aware	3485.6417 ± 1.5362	547.2368 ± 0.0000	553.8716 ± 0.0000
SFCLI-Net + SicTTA	3614.5238 ± 1.6428	583.4572 ± 0.0000	579.6425 ± 0.0000
Ours	**2252.1050 ± 1.3247**	**114.4042 ± 0.0000**	**380.9202 ± 0.0000**

**Table 26:** Interpolation performance on external liver CT and COVID-19 CT datasets.

Dataset	PSNR (dB)	SSIM	LPIPS	EdgeSSIM
Liver CT Dataset	38.7054 ± 0.25	0.9655 ± 0.0025	0.0611 ± 0.0018	0.8589 ± 0.0032
COVID-19 CT Dataset	38.1579 ± 0.32	0.9588 ± 0.0028	0.0468 ± 0.0021	0.8824 ± 0.0029

**Table 27:** Segmentation performance on external liver CT and COVID-19 CT datasets.

Dataset	Dice	IoU	Precision	Recall
Liver CT Dataset	0.9653 ± 0.0042	0.9360 ± 0.0051	0.9684 ± 0.0038	0.9647 ± 0.0045
COVID-19 CT Dataset	0.9046 ± 0.0053	0.8309 ± 0.0062	0.9169 ± 0.0049	0.8970 ± 0.0058

**Table 28:** Static model complexity metrics on external datasets.

Dataset	Parameters (M)	Memory (MB)	FLOPs (G)
Liver CT Dataset	33.3589 ± 0.0000	2252.1050 ± 1.0532	114.4042 ± 0.0000
COVID-19 CT Dataset	33.3589 ± 0.0000	2253.6519 ± 0.9849	114.4042 ± 0.0000

**Table 29:** Dynamic inference performance metrics on external datasets.

Dataset	Avg_Infer_Time (ms)	Avg_FPS	Model Size (MB)
Liver CT Dataset	56.6527 ± 1.25	35.3148 ± 0.35	380.9202 ± 0.0000
COVID-19 CT Dataset	56.2045 ± 0.98	35.5976 ± 0.42	380.9202 ± 0.0000

## Data Availability

Publicly available datasets were analyzed in this study. The datasets used in this work are listed as follows: 1. The primary Medical Segmentation Decathlon (MSD) datasets (Task02_Heart and Task06_Lung) are available at: http://medicaldecathlon.com/dataaws/. 2. 3D Liver Tumor CT Dataset for generalization evaluation: https://www.kaggle.com/datasets/prathamgrover/3d-liver-segmentation (accessed on 10 May 2026). 3. COVID-19 Chest CT Scans Dataset for generalization evaluation: https://www.kaggle.com/datasets/andrewmvd/covid19-ct-scans (accessed on 11 May 2026).

## Abbreviations

The following abbreviations are used in this manuscript:

TASC-SwinMT	Task-Adaptive Synergistic Cross-Task Swin Multi-Task Framework
TALA	Task-Aware Lightweight Adapter
MSTAF	Multi-Scale Task Alignment Fusion
CTCI	Cross-Task Collaborative Interaction
CT	Computed Tomography
MRI	Magnetic Resonance Imaging
BN	Batch Normalization
CNN	Convolutional Neural Network
FFT	Fast Fourier Transform
iFFT2D	Inverse Two-dimensional Fast Fourier Transform
BCE	Binary Cross-Entropy
MSE	Mean Squared Error
Dice	Dice Coefficient
EdgeSSIM	Edge-based Structural Similarity Index Measure
LPIPS	Learned Perceptual Image Patch Similarity
PSNR	Peak Signal-to-Noise Ratio
SSIM	Structural Similarity Index Measure
IoU	Intersection over Union
TCS	Temporal Consistency Score
MSD	Medical Segmentation Decathlon
MTL	Multi-Task Learning
SOTA	State-of-the-Art
MoE	Mixture of Experts
GradNorm	Gradient Normalization
DWA	Dynamic Weight Average
GPU	Graphics Processing Unit
FP16	Mixed precision training
FLOPs	Floating Point Operations
FPS	Frames Per Second
